# A Mechanistic Study on Iron-Based Styrene Aziridination: Understanding Epoxidation via Nitrene Hydrolysis

**DOI:** 10.3390/molecules29153470

**Published:** 2024-07-24

**Authors:** Dóra Lakk-Bogáth, Patrik Török, Dénes Pintarics, József Kaizer

**Affiliations:** Research Group of Bioorganic and Bio-Coordination Chemistry, University of Pannonia, H-8201 Veszprém, Hungary; lakkd@almos.uni-pannon.hu (D.L.-B.); patriktrk6@gmail.com (P.T.); pintarics.denes@gmail.com (D.P.)

**Keywords:** aziridination, epoxidation, iron, catalysis, kinetics

## Abstract

Transition-metal-catalyzed nitrene transfer reactions are typically performed in organic solvents under inert and anhydrous conditions due to the involved air and water-sensitive nature of reactive intermediates. Overall, this study provides insights into the iron-based ([Fe^II^(PBI)_3_](CF_3_SO_3_)_2_ (1), where PBI = 2-(2-pyridyl)benzimidazole), catalytic and stoichiometric aziridination of styrenes using PhINTs ([(*N*-tosylimino)iodo]benzene), highlighting the importance of reaction conditions including the effects of the solvent, co-ligands (*para*-substituted pyridines), and substrate substituents on the product yields, selectivity, and reaction kinetics. The aziridination reactions with **1**/PhINTs showed higher conversion than epoxidation with 1/PhIO (iodosobenzene). However, the reaction with PhINTs was less selective and yielded more products, including styrene oxide, benzaldehyde, and 2-phenyl-1-tosylaziridine. Therefore, the main aim of this study was to investigate the potential role of water in the formation of oxygen-containing by-products during radical-type nitrene transfer catalysis. During the catalytic tests, a lower yield was obtained in a protic solvent (trifluoroethanol) than in acetonitrile. In the case of the catalytic oxidation of *para*-substituted styrenes containing electron-donating groups, higher yield, TON, and TOF were achieved than those with electron-withdrawing groups. Pseudo-first-order kinetics were observed for the stoichiometric oxidation, and the second-order rate constants (*k*_2_ = 7.16 × 10^−3^ M^−1^ s^−1^ in MeCN, 2.58 × 10^−3^ M^−1^ s^−1^ in CF_3_CH_2_OH) of the reaction were determined. The linear free energy relationships between the relative reaction rates (log*k*_rel_) and the total substituent effect (TE, 4R-PhCHCH_2_) parameters with slopes of 1.48 (MeCN) and 1.89 (CF_3_CH_2_OH) suggest that the stoichiometric aziridination of styrenes can be described through the formation of a radical intermediate in the rate-determining step. Styrene oxide formation during aqueous styrene aziridination most likely results from oxygen atom transfer via in situ iron oxo/oxyl radical complexes, which are formed through the hydrolysis of [Fe^III^(N•Ts)] under experimental conditions.

## 1. Introduction

The formation of carbon–oxygen and carbon–nitrogen bonds with attractive selectivity is one of the important challenges facing synthetic chemists. Both aziridination and epoxidation are important reactions in organic chemistry that form three-membered rings.

Aziridines are nitrogen-containing analogs of epoxides, which have attracted the interest of synthetic organic chemists only in the past 30 years. Several reviews deal with their synthesis [1,2,3,4,5], physical properties [6,7], biological properties [8,9], reactions [10,11], and their comparison with epoxides [8,12]. 

The formation of carbon–nitrogen bonds is definitely crucial in the synthesis of biologically active molecules [13,14]. Nitrene transfer reactions involve the transfer of a nitrene moiety (a nitrogen atom with a lone pair of electrons) from one molecule to another, resulting in the formation of a new carbon–nitrogen bond. Nitrene transfer reactions offer an alternative to heteroatom coupling methods that require the prefunctionalization of substrates but typically involve metal catalysts, often containing expensive and toxic metals such as Rh, Ru, and Pd [15,16,17,18,19]. Therefore, there has been a push to develop greener catalytic systems based on more abundant and non-toxic metals, such as Co, Cu, and especially Fe [16,20,21,22,23,24,25,26,27]. It is generally believed that iron-catalyzed nitrene transfer reactions involve high-valent iron–imido species, similar to oxygenase enzymes [28]. In recent years, many iron–imido and iron–imidyl complexes have been isolated [29,30,31,32,33,34], but their catalytic activity has only been investigated in a few cases.

A mononuclear nonheme iron(IV)–imido complex [(N_4_Py)Fe^IV^(NTs)]^2+^ (N_4_Py: *N*,*N*-bis(2-pyridylmethyl)-*N*-bis(2-pyridyl)-methylamine) was synthesized and then inquired into in nitrene transfer reactions and C-H bond activation reactions [29,35,36,37]. Nam and his colleagues described the first example of a mononuclear nonheme iron(V)–imido complex carrying a tetraamido macrocyclic ligand (TAML), [(TAML)Fe^V^(NTs)]^−^ [28]. After spectroscopically determining the S = 1/2 iron(V) oxidation state of this complex, its reactivity for C-H bond functionalization and nitrene transfer reactions was investigated.

During our previous research, we investigated the catalytic and stoichiometric oxidation of styrene derivatives utilizing a nonheme complex ([Fe^II^(PBI)_3_](CF_3_SO_3_)_2_) as a catalyst and PhIO as an oxidant [38]. The catalytic activity was moderate, but the reaction proved to be selective, yielding epoxides as the main product (the benzaldehyde by-product was below 1% in all cases). The reactions carried out with *para*-substituted styrenes led to the reaction mechanism in which the rate-determining step involves an uncoordinated electron transfer mechanism through the formation of the radicaloid benzylic radical intermediate. The results of the stoichiometric epoxidation reactions performed with the Fe^III^(OIPh) intermediate produced in situ supported our theory regarding catalysis.

As a continuation of this work, we investigated the catalytic and stoichiometric oxidation of various alkenes (*cis*-cyclooctene, *cis*-stilbene, and *trans*-stilbene) using a [Fe^II^(PBI)_3_](CF_3_SO_3_)_2_ (**1**) catalyst and PhI(OAc)_2_ oxidant in the presence of pyridine derivatives [39]. The performed experiments suggest that pyridine additives have a significant effect on the catalytic activity of the oxygen transfer processes, likely by their coordination as an equatorial co-ligand to the iron center. The oxidizing agent can be an electrophilic [(PBI)_2_(4R-Py)Fe^IlI^OIPh]^3+^ intermediate, the formation of which is indicated by the highest increase rate achieved during epoxidation reactions carried out in the presence of pyridines containing an electron-withdrawing group. During UV-vis measurements, if the solvent molecule was replaced by pyridines, the corresponding [(PBI)_2_[(4R-Py)Fe^III^(OIPh)]^3+^ species were formed and a linear correlation was found between the energy of the characteristic absorption bands (λ_max_^−1^ at 700–760 nm) and the Hammett substituent constants (σ_p_), which indicates that the hypsochromic shift (blue shift) observed in the absorption bands is indirectly related to the electronic effect of the co-ligands and the electrophilicity of the active intermediate. Since iodosobenzene (PhIO) is isoelectronic with [(*N*-tosylimino)iodo]benzene (PhINTs), our goal was to carry out catalytic aziridination (*N*-transfer) reactions with **1** as a catalyst and PhINTs as an oxidant, similar to the previously published epoxidation system. Many cases of catalytic olefin aziridination with iron and manganese complexes yielded epoxides next to the desired aziridine products. Although this work acknowledges the potential role of water, no in-depth studies on the formation of such side-products are reported. The overall reactivity of (radical-type) metal–nitrene complexes in the presence of water remains rather poorly understood [16]. Therefore, this study aims to investigate the potential role of water in forming oxygen-containing side-products during radical-type nitrene transfer catalysis and explores the mechanism of this process to circumvent this issue in iron-catalyzed nitrene transfer in the presence and absence of water.

## 2. Results and Discussion

**Scheme 1:**
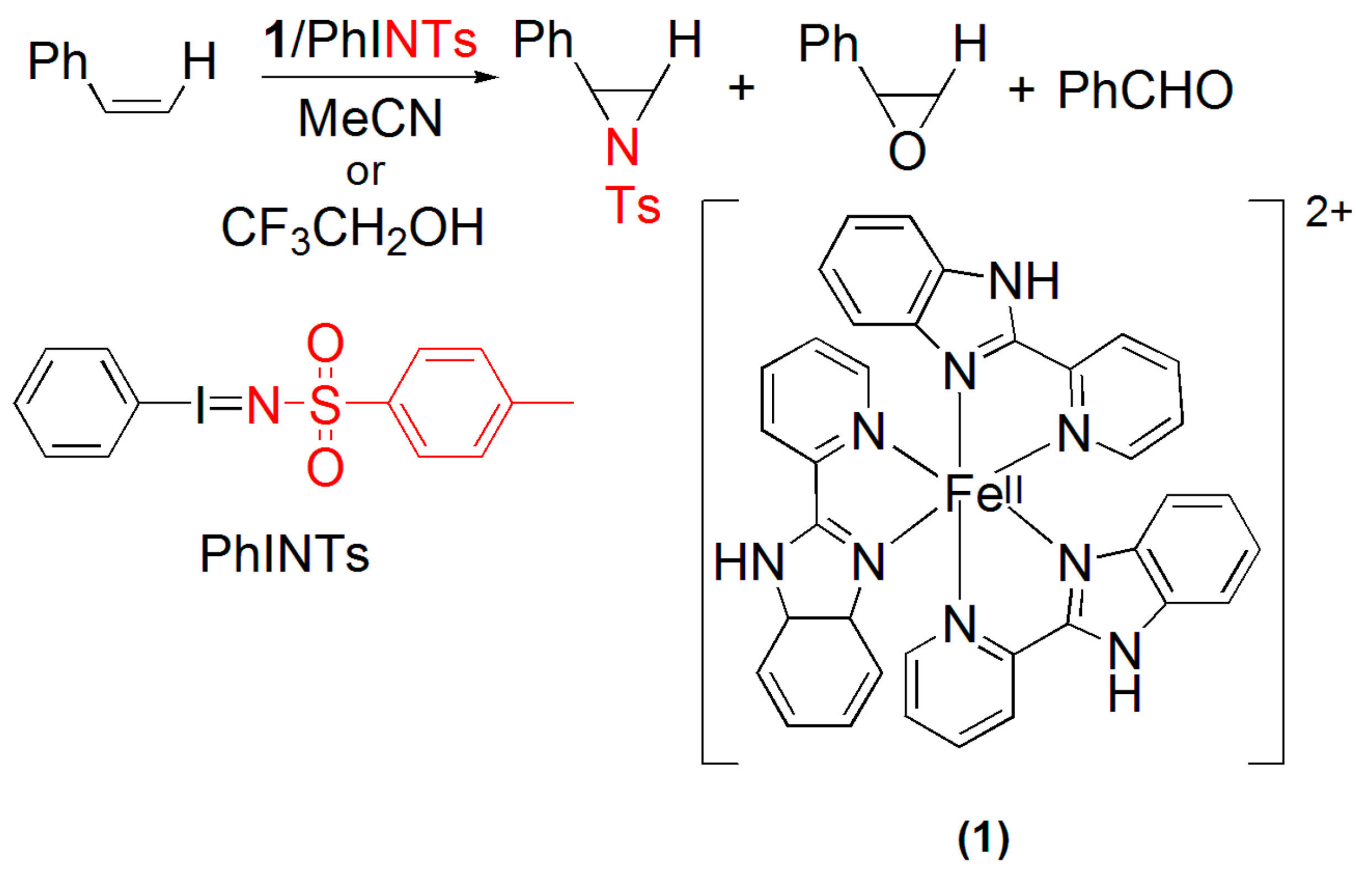
Products formed during stoichiometric and catalytic aziridination of styrene and structures of PhINTs and [Fe^II^(PBI)_3_(CF_3_SO_3_)_2_] (**1**).

In order to gain insights into the mechanism of aziridination, we investigated the reaction of styrene with a previously characterized iron complex (**1**) and PhINTs (Scheme 1).

### 2.1. Catalytic Aziridination of Styrenes

**Figure 1:**
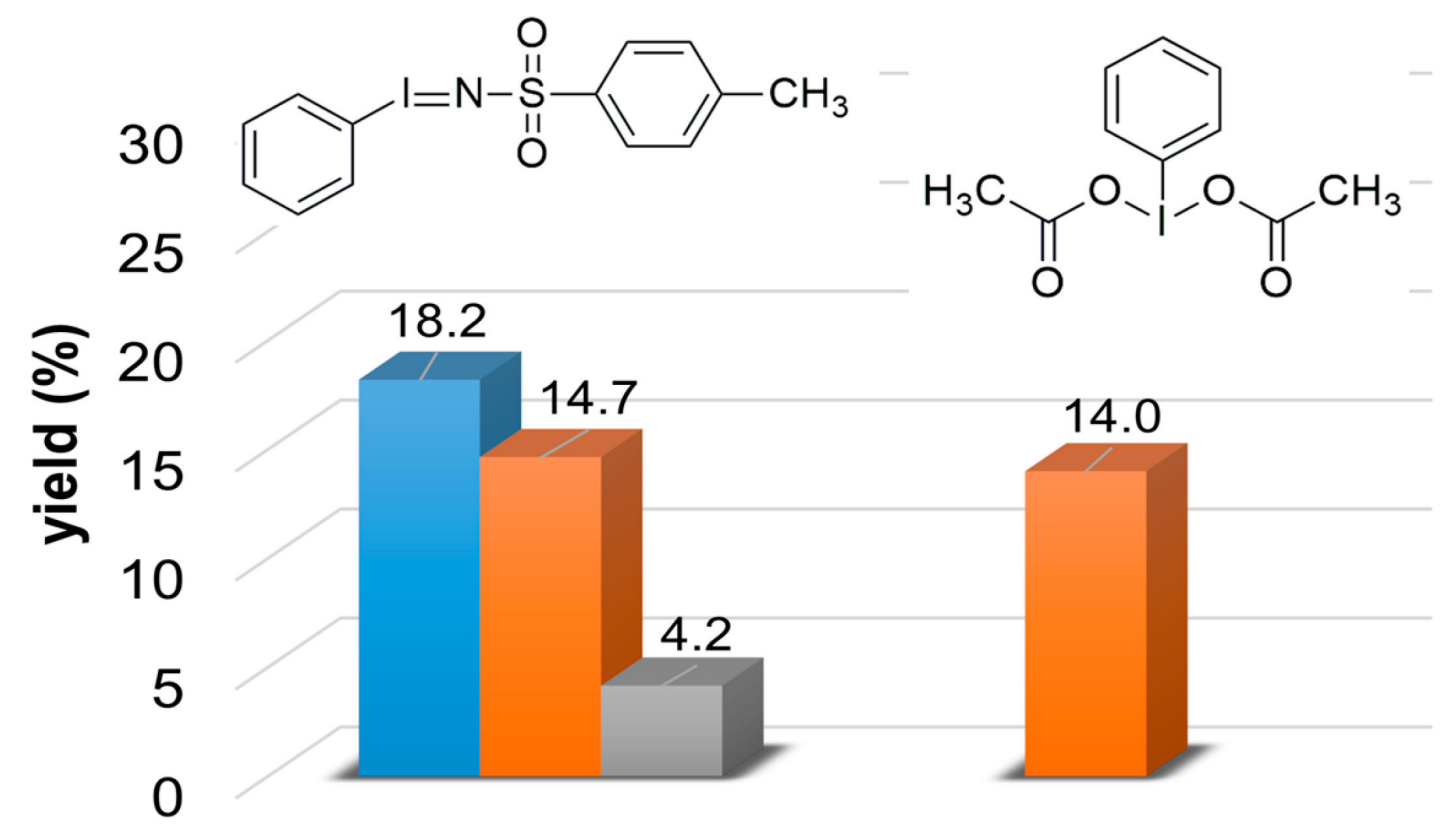
The yield of products for the catalytic oxidation of styrene at 323 K in MeCN with PhINTs and PhI(OAc)_2_ (PhIO): benzaldehyde (▪), styrene oxide (▪), and 2-phenyl-1-tosylaziridine (▪). [1]_0_ = 1 × 10^−3^ M, [PhIO or PhINTs]_0_ = 1 × 10^−1^ M, and [styrene]_0_ = 3 × 10^−1^ M under air.

As a preliminary experiment, the catalytic aziridination of styrene was carried out at a catalyst/oxidant/substrate ratio of 1:100:300 in distilled acetonitrile solvent at 323 K for 4 h like the epoxidation of styrenes under optimal conditions [33]. Compared to the epoxidation of styrene with iodosobenzene (PhIO), a higher conversion was observed when the oxidation reaction of styrene was carried out with PhINTs, but when examining the composition of the product mixture, the reaction was not selective, and three products were formed, where the yield of the sryrene oxide (SO, 14.7%) was almost the same as before (14%), but benzaldehyde (Bz, 18.2%) and 2-phenyl-1-tosylaziridine (SNTs, 4.2%) as well as the decay products of PhNTs, PhI, and TsNH_2_ were also formed (Figure 1). Since almost the same results were obtained under argon and air, the question arises as to where the oxygen of epoxide and benzaldehyde comes from (traces of water in the solvent, oxidizing agent, or from the air). Therefore, it is crucial to investigate how these transformations result in the undesired oxygenated products. Similar results were obtained in catalytic olefin aziridination with iron and manganese complexes using hypervalent iminoiodinanes as nitrene precursors, which yielded epoxides next to the desired aziridine products, where the potential role of water in the formation of such side-products was proposed. However, the overall reactivity of (radical-type) metal–nitrene complexes in the presence of water is still rather poorly understood [40]. It can be assumed that the iron–nitrene/nitrene radical complexes may suffer from hydrolysis in a wet solvent, which may result in oxo/oxyl radical complexes active in OAT (oxygen atom transfer).

**Figure 2:**
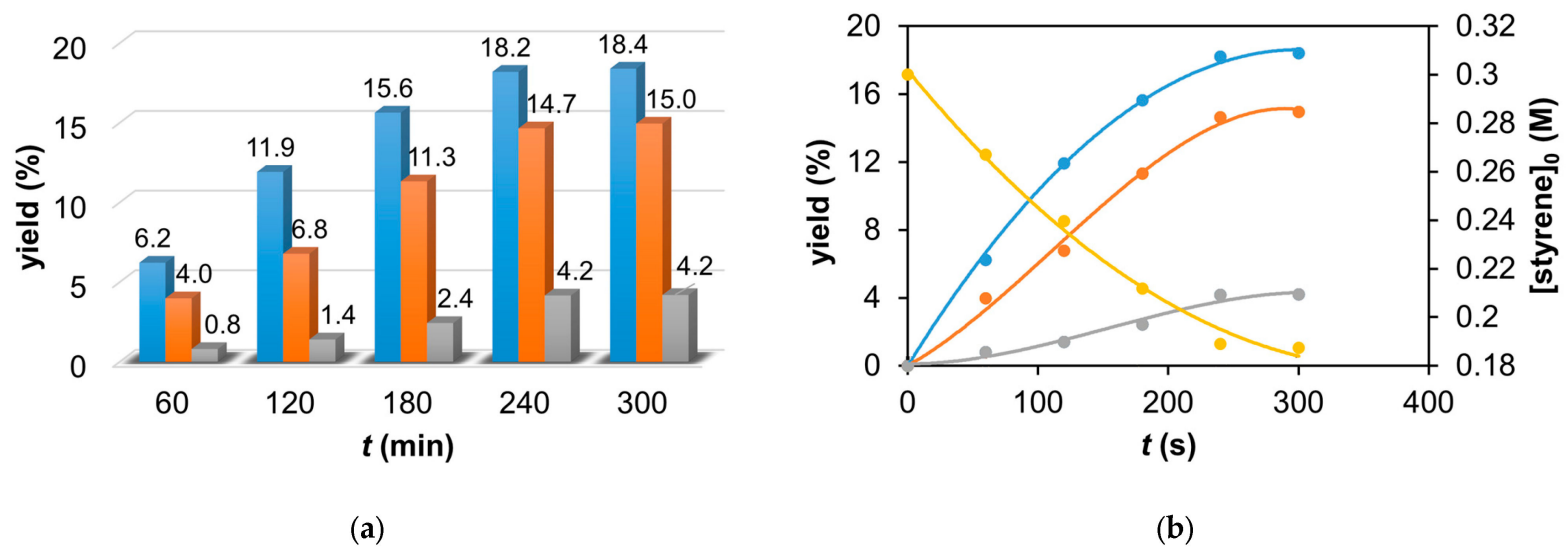
The catalytic oxidation of styrene at 323 K in MeCN at different times. (**a**) The yields of benzaldehyde (▪), styrene oxide (▪), and 2-phenyl-1-tosylaziridine (▪) for this reaction. (**b**) The yields of benzaldehyde (▪), styrene oxide (▪), and 2-phenyl-1-tosylaziridine (▪) and the decrease in the styrene concentration (▪) as a function of time for the catalytic oxidation of styrene at 323 K in MeCN. [1]_0_ = 1 × 10^−3^ M, [PhINTs]_0_ = 1 × 10^−1^ M, and [styrene]_0_ = 3 × 10^−1^ M under air.

**Figure 3:**
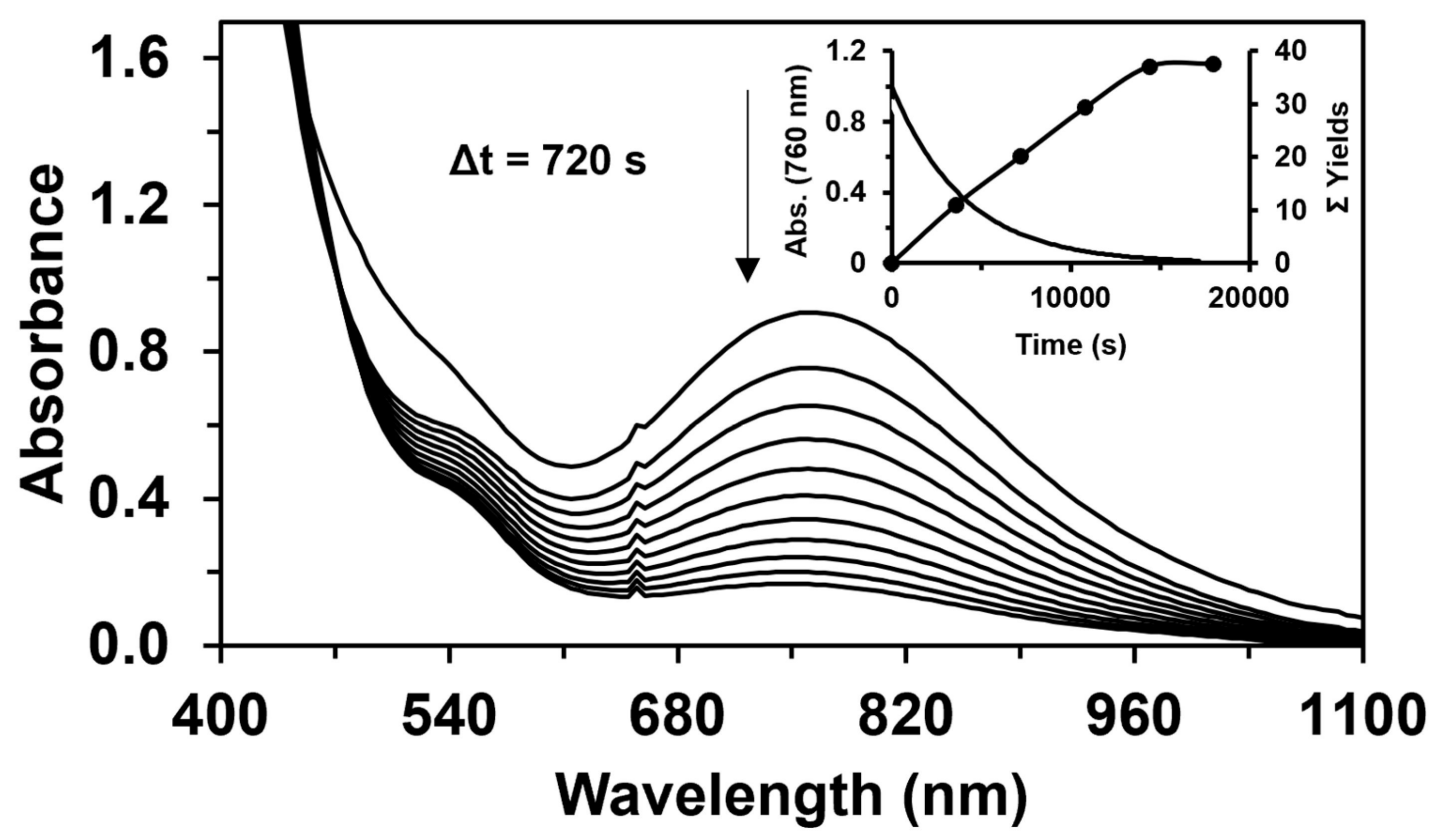
The self-decay of **1** in the absence of a substrate at 323 K in MeCN. [1]_0_= 1 × 10^−3^ M; [PhINTs]_0_ = 1.2 × 10^−3^ M. Inset: the change in the absorbance of the **1**/PhINTs adduct at 760 nm and the total yield as a function of time for the catalytic oxidation of styrene at 323 K in MeCN. [1]_0_ = 1 × 10^−3^ M, [PhINTs]_0_ = 1 × 10^−1^ M, and [styrene]_0_ = 3 × 10^−1^ M.

In order to achieve the maximum yield of the products (Figure 2a), the oxidation of styrene was monitored over time (Figure 2b), and the highest values (Bz, 18.2%; SO, 14.7%; and NTs, 4.2%) were obtained after 4 h (Table 1). At the highest conversion value (37% based on the oxidant), which corresponds to a turn-over number (TON) of 37, the epoxide-to-aziridine ratio (SO/SNTs) was 3.5. Figure 3 shows a control experiment monitored by UV-vis measurements in the absence of a substrate, which proves the stability of the intermediate, and its disappearance may correlate with the formation of products.

**Figure 4:**
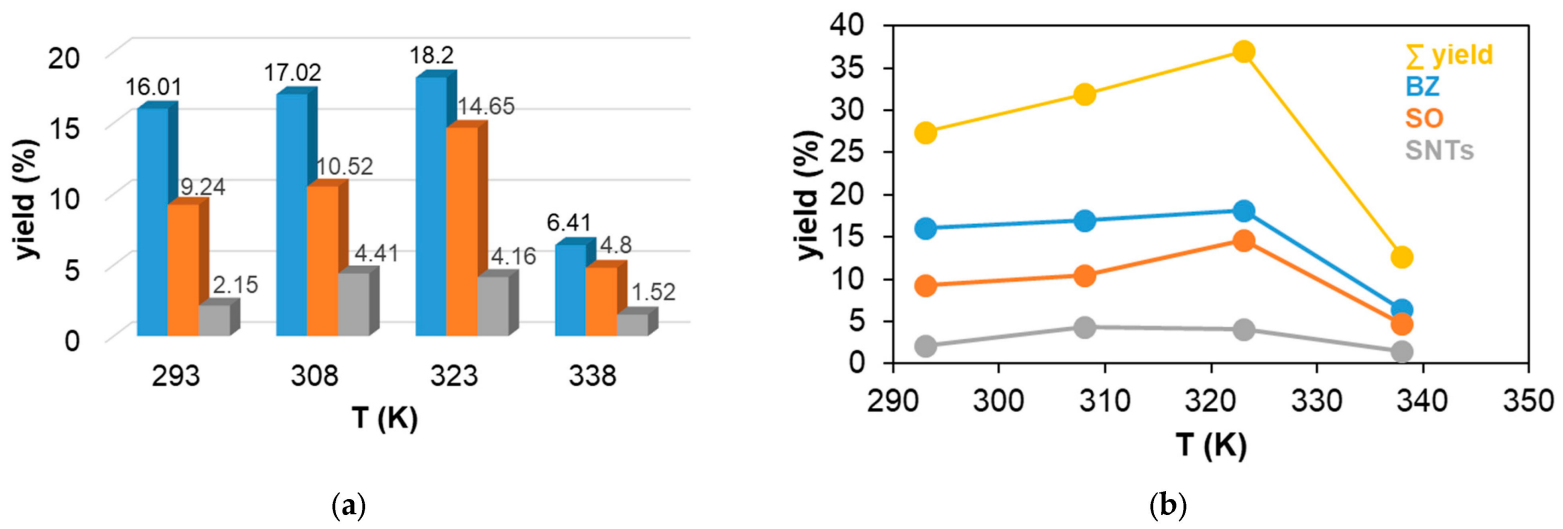
The catalytic oxidation of styrene at different temperatures in MeCN. (**a**) The yields of Bz, benzaldehyde (▪); SO, styrene oxide (▪); and SNTs, 2-phenyl-1-tosylaziridine (▪) for this reaction. (**b**) The yields of Bz (▪), SO (▪), and SNTs (▪) and the total yield (▪) as a function of temperature for the catalytic oxidation of styrene in MeCN. [1]_0_= 1 × 10^−3^ M, [PhINTs]_0_ = 1 × 10^−1^ M, and [styrene]_0_ = 3 × 10^−1^ M under air.

In addition to the above conditions, the reaction was also carried out at 293, 308, and 338 K (Figure 4a). Based on the obtained results, it can be said that the highest product yield values were obtained at 323 K (Figure 4b). Looking at the ratio of the products, it can also be said that the effect of temperature on selectivity is negligible, and the formation of aziridine is not significant in the tested temperature range. The low yields obtained at 338 K are probably explained by the decomposition of the catalyst and/or the PhINTs (Table 2).

**Figure 5:**
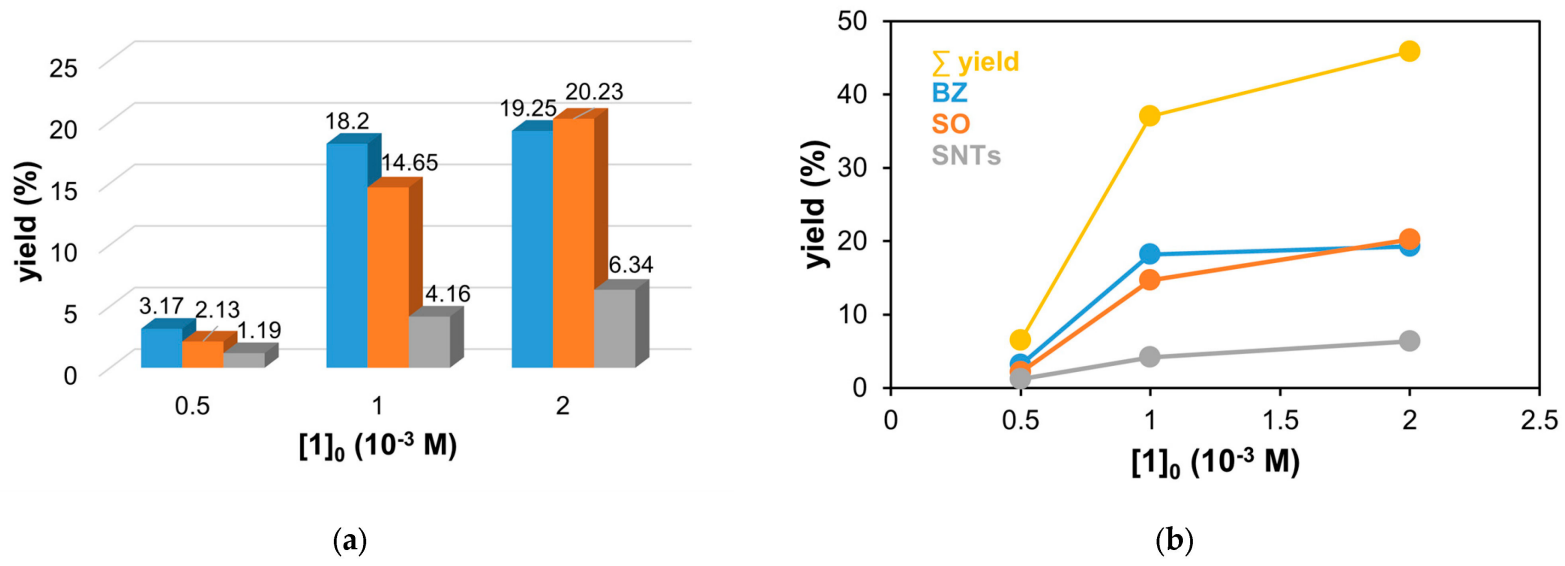
The catalytic oxidation of styrene at different metal concentrations in MeCN at 323 K. (**a**) The yields of Bz, benzaldehyde (▪); SO, styrene oxide (▪); and SNTs, 2-phenyl-1-tosylaziridine (▪) for this reaction. (**b**) The yields of benzaldehyde (▪), styrene oxide (▪), and 2-phenyl-1-tosylaziridine (▪) and the total yield (▪) as a function of the iron concentration for the catalytic oxidation of styrene in MeCN at 323 K. [PhINTs]_0_ = 1 × 10^−1^ M; [styrene]_0_ = 3 × 10^−1^ M under air.

In order to optimize catalyst performance, the aziridination of styrene was also carried out with varying catalyst/styrene ratios (Figure 5a, Table 3). Based on the composition of the product mixture, it can be concluded that by changing the catalyst/substrate ratio from 1:300 to 2:300, the amount of benzaldehyde remained almost unchanged, while the amounts of both epoxide and aziridine increased by approximately one and a half times (Figure 5b). However, the epoxide/aziridine ratio did not change (~3–4). Since the amount of benzaldehyde does not depend on the concentration of the complex, it is likely that its formation can be described by a side reaction.

The effect of the substituents in the *para*-position of the aromatic ring (4R-Ph) and on the vinyl group (-CH=CH_2_) of the styrene was also investigated in both acetonitrile and trifluoroethanol (Table 4 and Table 5). Using α-methylstyrene as a substrate in acetonitrile, acetophenone and the corresponding epoxide and aziridine were obtained as products in a ratio of 5:8:1, but with much lower yields (acetophenone, 3.19%; SO, 5.16%; SNTs, 0.61%) than in the case of styrene under the same conditions. It is conceivable that the decreases in product yields can be explained by steric hindrance due to the α-methyl group.

**Figure 6:**
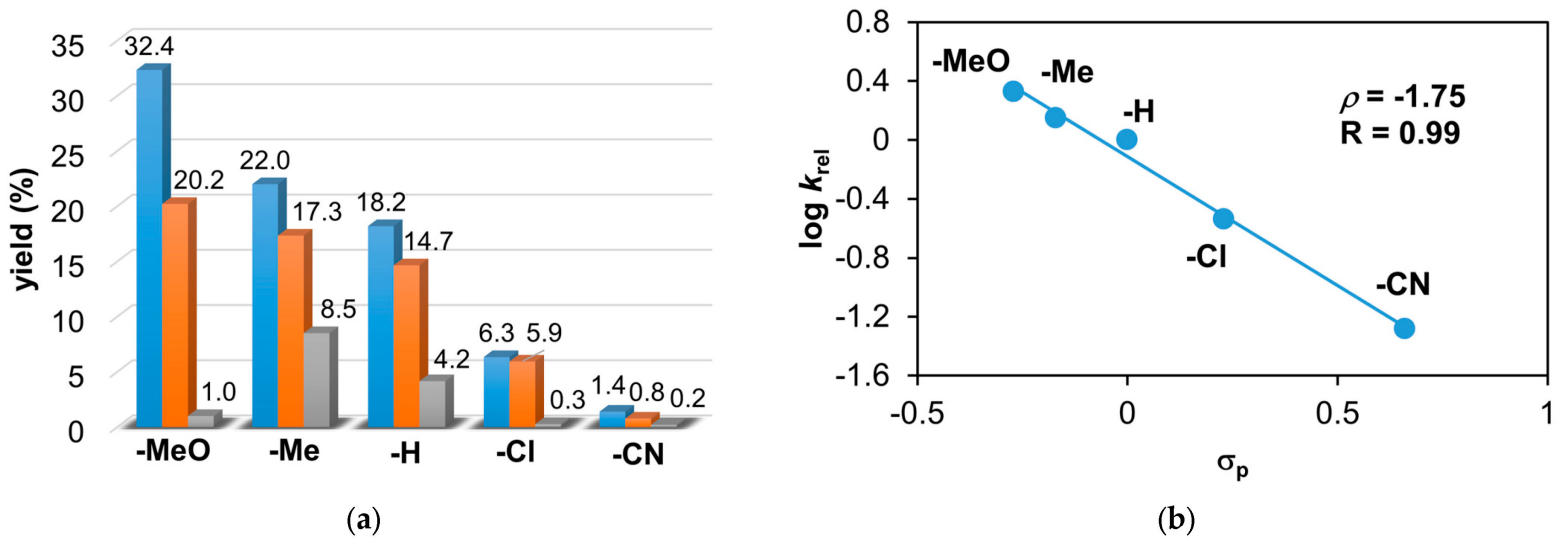
The catalytic oxidation of different *para*-substituted styrenes at 323 K in MeCN. (**a**) The yields of products for this reaction: aldehyde (▪), styrene oxide (▪), and tosylaziridine (▪). (**b**) The plot of log*k*_rel_ against σ_p_ of *para*-substituted styrenes. [1]_0_ = 1 × 10^−3^ M, [PhINTs]_0_ = 1 × 10^−1^ M, and [styrene]_0_ = 3 × 10^−1^ M under air.

**Figure 7:**
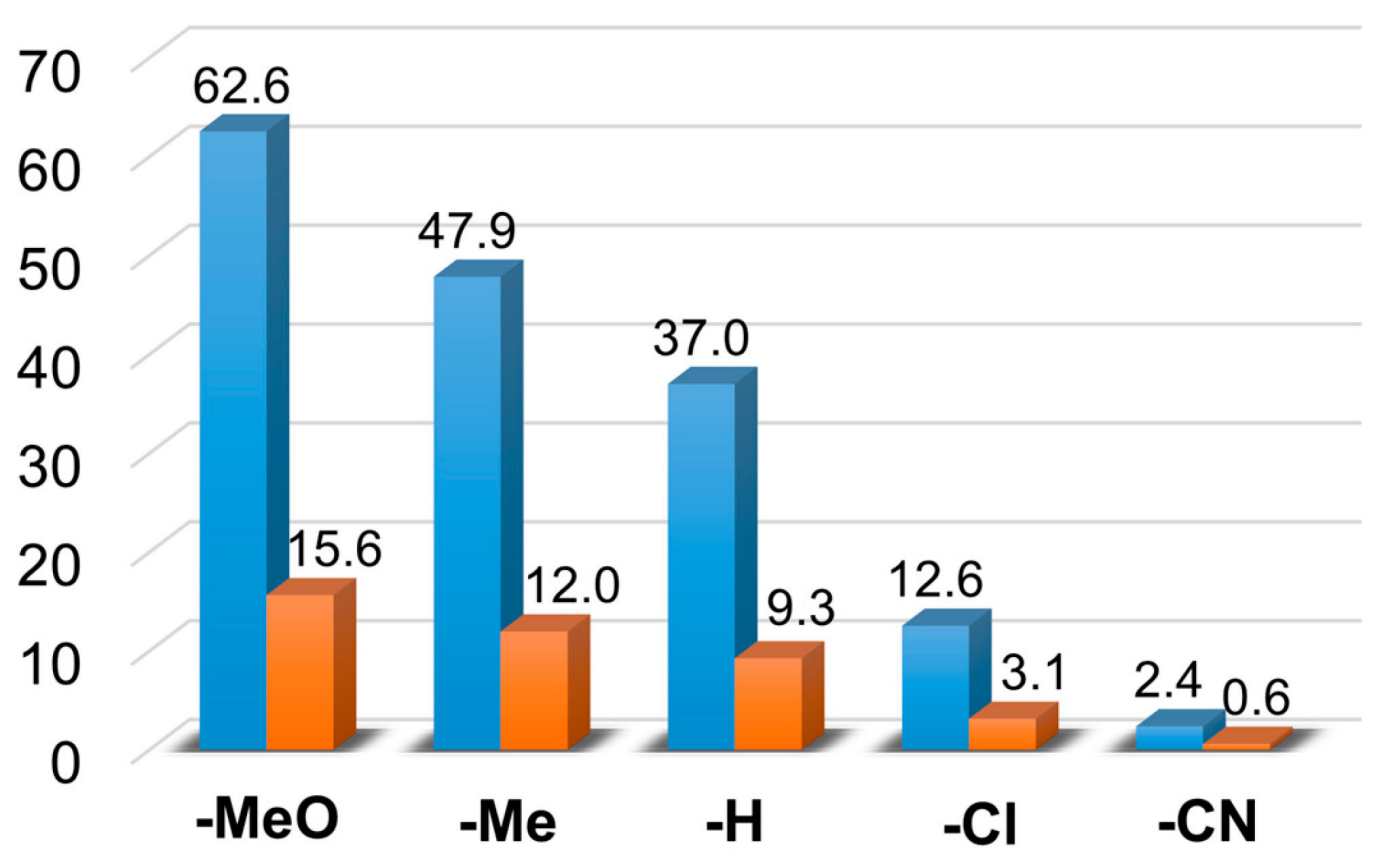
The TON (▪) and TOF (▪) (1/h) values for the catalytic oxidation of *para*-substituted styrenes at 323 K in MeCN. [1]_0_ = 1 × 10^−3^ M, [PhINTs]_0_ = 1 × 10^−1^ M, and [styrene]_0_ = 3 × 10^−1^ M under air.

Regarding the catalytic oxidation of various *para*-substituted styrenes, the highest yields, TON and TOF were observed in the case of styrenes containing an electron-donating group (4MeO-S, 62.56%, 62.56, and 15.64 h^−1^, respectively), while the lowest yields, TON and TOF were observed in those containing an electron-withdrawing group (4CN-S, 2.38%, 2.38, and 0.59 h^−1^, respectively) (Table 4 and Figure 6a and Figure 7). In all cases, benzaldehyde was the main product, the formation of which can be deduced from the epoxide [41] via a side reaction. The ratio of epoxide/aziridine products is relatively high in all cases, and the highest value (~20) was observed for 4MeO-S. When plotting the relative reactivity values (*k*_rel_ = log(X_f_/X_i_)/log(Y_f_/Y_i_), where X_i_ and X_f_ are the initial and final concentrations of 4R-styrene, and Y_i_ and Y_f_ are the initial and final concentrations of styrene) of *para*-substituted styrenes as a function of σ_p_, the *ρ* value was −1.75, suggesting that the behavior of the oxidant generated from **1** and PhINTs is electrophilic (Figure 6b). To address the involvement of the proposed nitrene radical complexes, we performed the aziridination reaction of styrene with **1** in the presence of 2,6-ditertbutyl-4-methylphenol as a radical scavenger under anaerobic conditions in MeCN. The addition of five equivalents of 2,6-ditertbutyl-4-methylphenol with respect to **1** completely inhibited the oxidation (aziridination) of styrene. The absence of products indicates that a radical-type intermediate is generated and subsequently trapped. Taken together, these experiments support the hypothesis that the Fe^III^(N•Ts) complex is formed and reacts with styrene directly to give aziridine as a product or with traces of water to form another reactive intermediate. The latter is most likely the Fe^IV^(O)/oxyl complex, which is responsible for the formation of styrene oxide.

**Figure 8:**
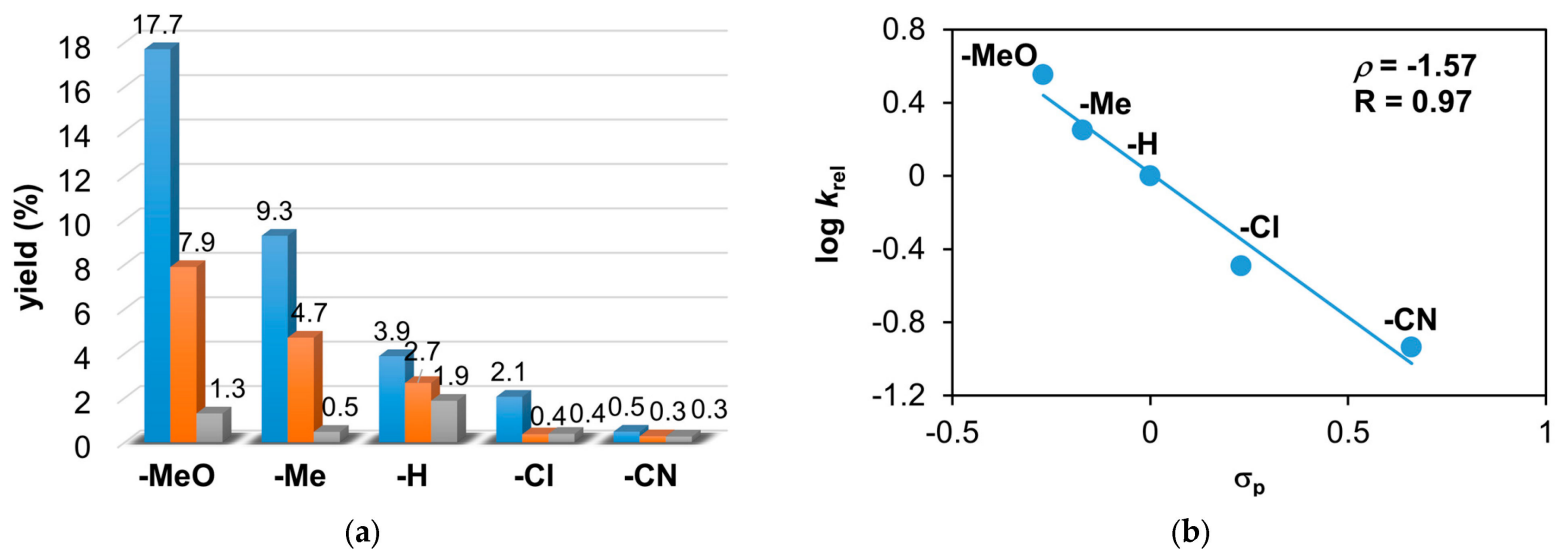
The catalytic oxidation of different *para*-substituted styrenes at 323 K in CF_3_CH_2_OH. (**a**) The yields of products for this reaction: aldehyde (▪), styrene oxide (▪), and tosylaziridine (▪), (**b**) The plot of log*k*_rel_ against σ_p_ of *para*-substituted styrenes. [1]_0_ = 1 × 10^−3^ M, [PhINTs]_0_ = 1 × 10^−1^ M, and [styrene]_0_ = 3 × 10^−1^ M under air.

**Figure 9:**
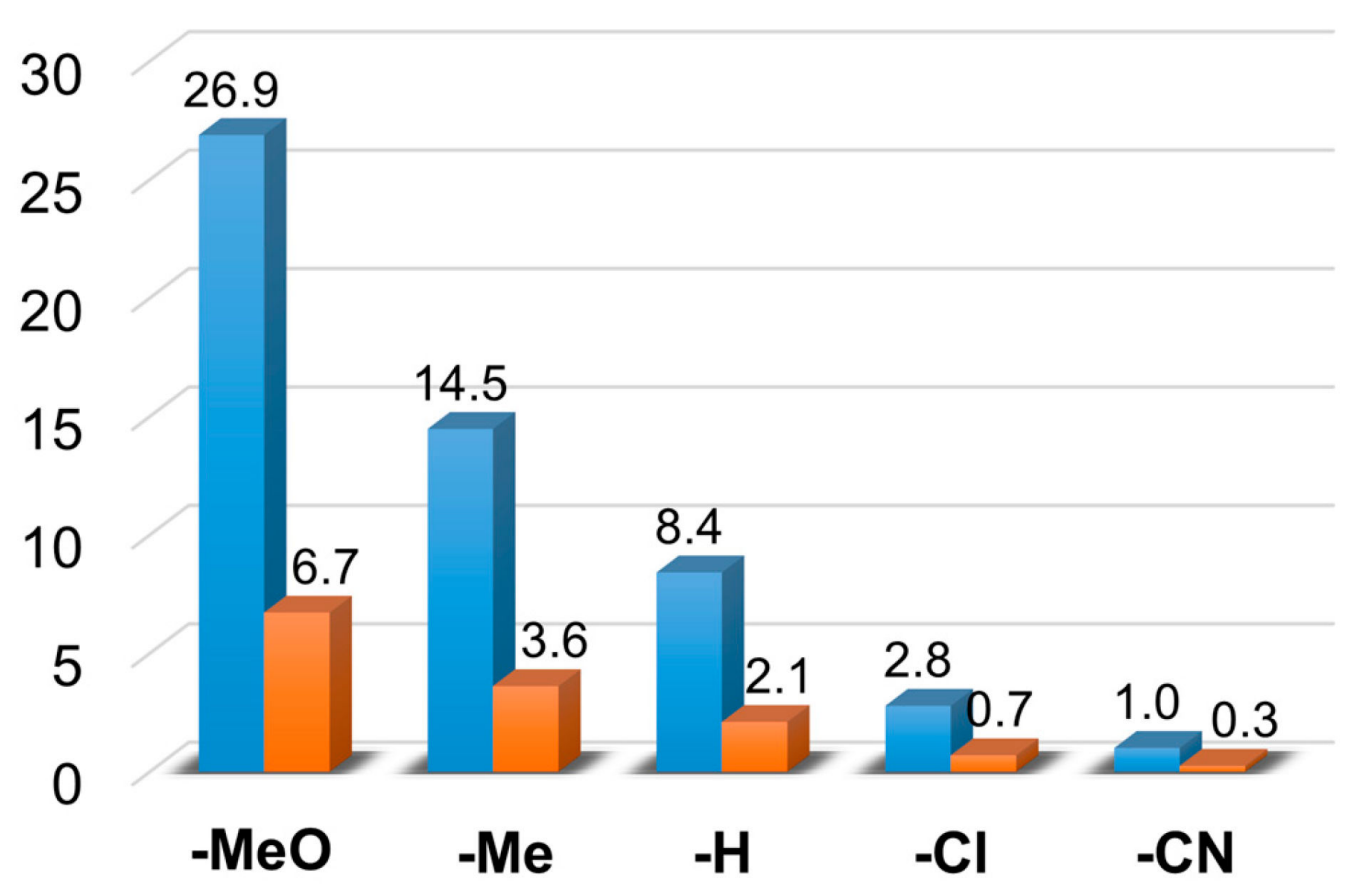
The TON (▪) and TOF (▪) (1/h) values for the catalytic oxidation of *para*-substituted styrenes at 323 K in CF_3_CH_2_OH. [1]_0_ = 1 × 10^−3^ M, [PhINTs]_0_ = 1 × 10^−1^ M, and [styrene]_0_ = 3 × 10^−1^ M under air.

The reactions were also carried out in the protic trifluoroethanol solvent, where much lower yields (Figure 8a; Table 5) and TON and TOF values (Figure 9) were obtained. In the case of all three products, the highest yield was observed in the case of the substrate containing an electron-donating group (4MeO-S, 26.89%, 26.89, and 6.72 h^−1^, respectively), as previously seen in acetonitrile. In all cases, benzaldehyde was the main product, and the ratio of epoxide/aziridine products was between 1 and 9. The highest value (~9) was observed for 4MeO-S. The *ρ* value obtained from the Hammett curve is −1.57 (Figure 8b), which is similar to the experiments performed in acetonitrile, indicating that the behavior of the oxidant derived from **1** and PhINTs is electrophilic.

**Scheme 2:**
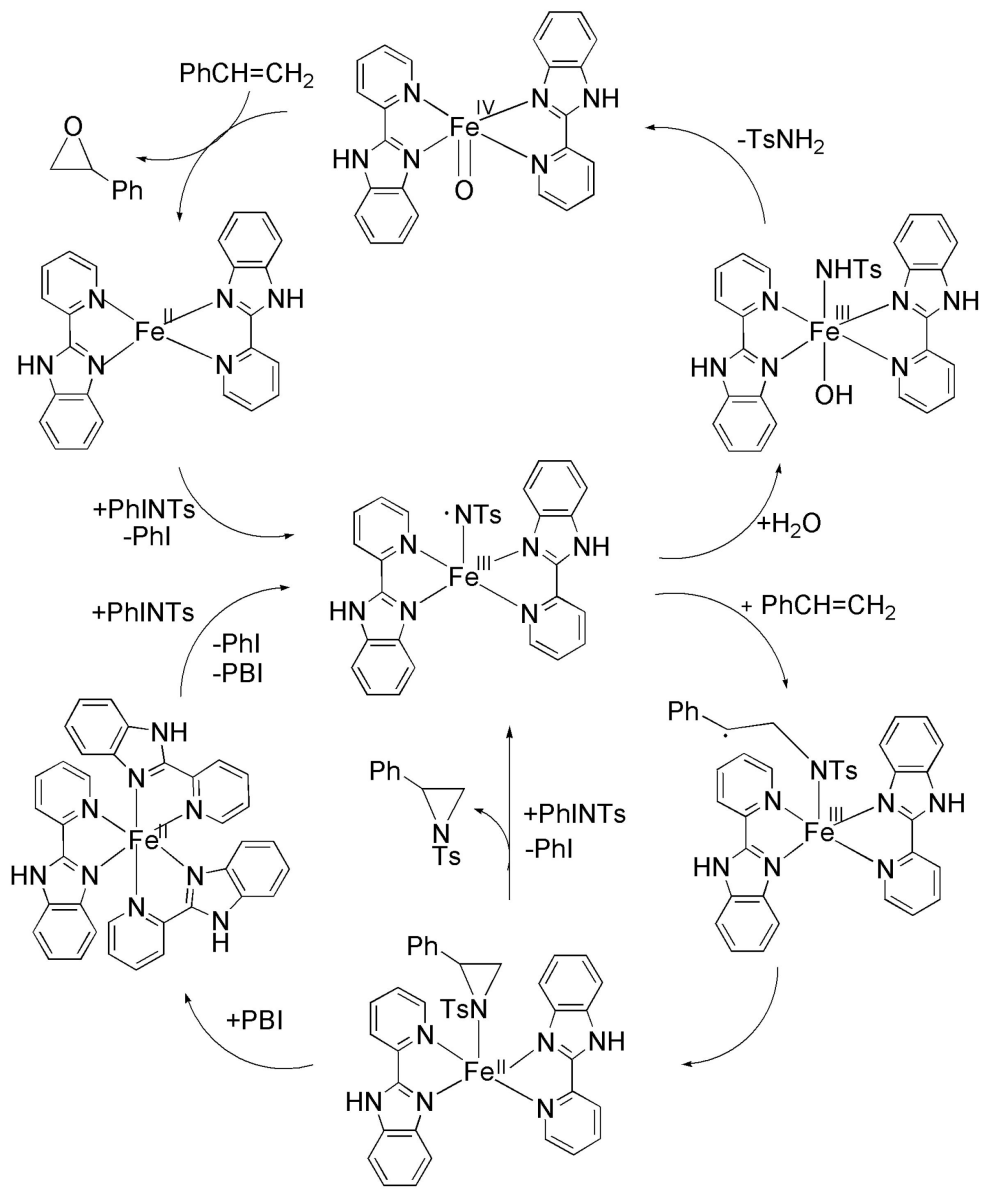
The proposed mechanisms for the Fe^III^(NTs)-mediated aziridination reactions.

**Figure 10:**
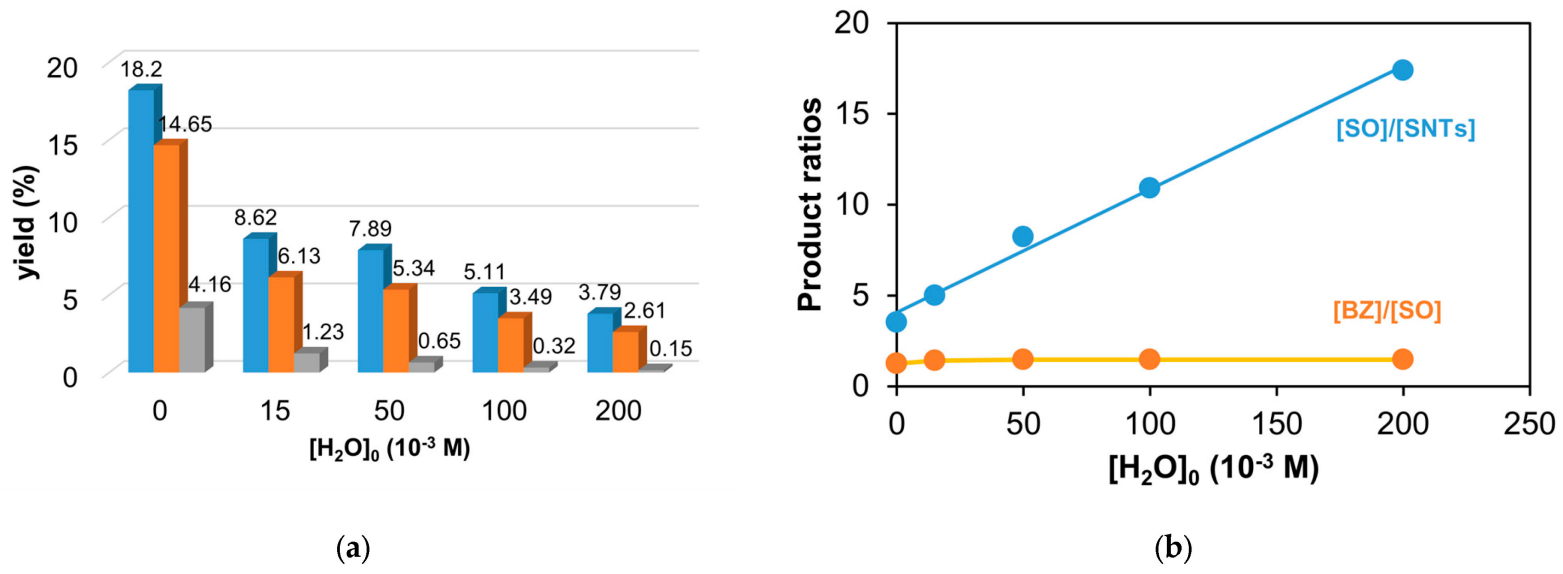
The effect of water for the catalytic oxidation of styrene. (**a**) The yields of products for this reaction (Bz, benzaldehyde (▪); SO, styrene oxide (▪); and SNTs, 2-phenyl-1-tosylaziridine (▪)). (**b**) The epoxide/aziridine ratio and the benzaldehyde/epoxide ratio as functions of the H_2_O concentration; [1]_0_ = 1 × 10^−3^ M, [PhINTs]_0_ = 1 × 10^−1^ M, and [styrene]_0_ = 3 × 10^−1^ M under air.

**Figure 11:**
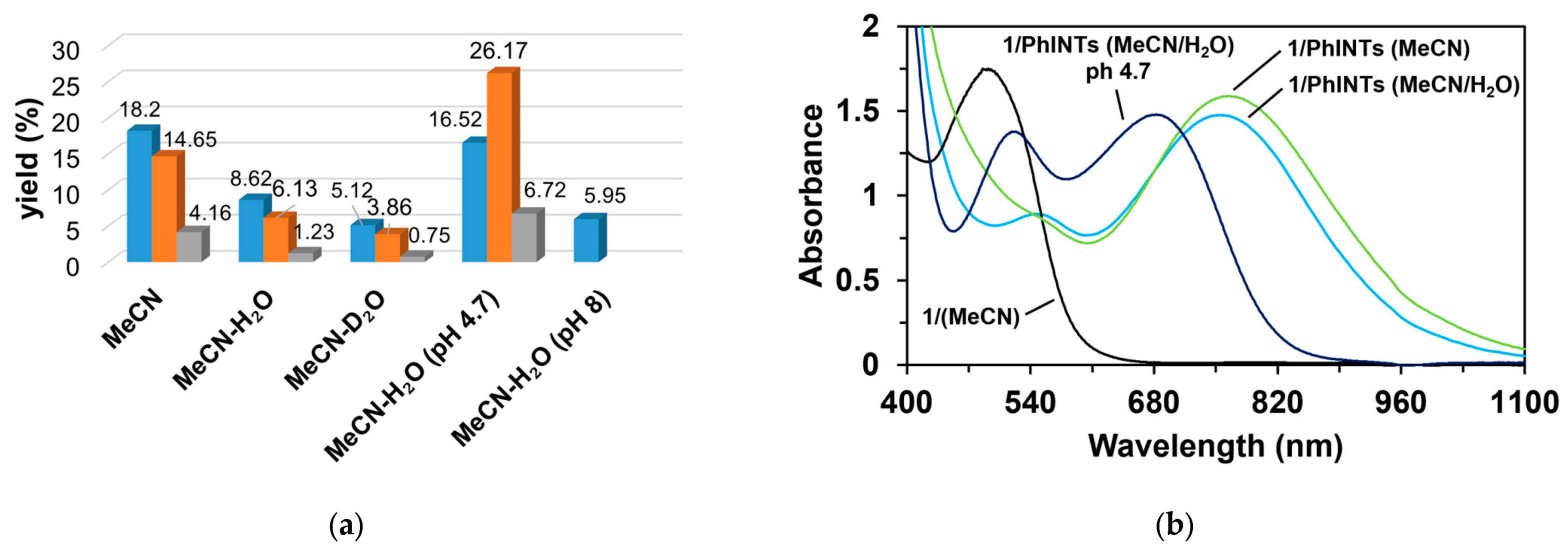
The effects of water, D_2_O, and the buffer for the catalytic oxidation of styrene. (**a**) The yields of products for this reaction: benzaldehyde (▪), styrene oxide (▪), and 2-phenyl-1-tosylaziridine (▪). (**b**) The change in wavelength due to water and the pH 4.7 buffer. [1]_0_ = 1 × 10^−3^ M, [PhINTs]_0_ = 1 × 10^−1^ M, [styrene]_0_ = 3 × 10^−1^ M, [H_2_O, D_2_O]_0_ = 1.5 × 10^−2^ M, and [buffer]_0_ = 2 × 10^−1^ mL under air.

Based on the results of our preliminary experiments, we came to the conclusion that the formation of the epoxide during the reaction may be explained by traces of water in the solvent (MeCN). Further experiments were carried out to find out the effect of water on the selectivity of the aziridination reaction and to explain the formation of the epoxide. By carrying out the reaction in acetonitrile by adding 15–200 equivalents of H_2_O (compared to **1**), we obtained the following results (Table 6): First of all, it can be established that even small amounts of water significantly reduce the product yields (Figure 10a). Looking at the product ratios, however, it is clearly visible that the ratio of SO/SNTs increases and shows a linear correlation with the increasing amount of water, while the Bz/SO ratio is almost constant (Figure 10b). After that, the aziridination of styrene was carried out in the presence of H_2_O and D_2_O under the same conditions. The SIE (solvent isotope effect) value calculated from the ratio of the products ([SO in H_2_O]/[SO in D_2_O] = 6.13%/3.86%) was around 1.6, which indicates the participation of water and/or H^+^ in the undesired epoxide formation (Figure 11a, Table 6). These results show that the epoxidation of styrene via Fe^IV^(O)/Fe^III^O•—formed by the hydrolysis of Fe^III^(N•Ts)—is feasible under the experimental conditions. The product selectivity of the nitrene transfer reaction is therefore dependent on the relative rates of the hydrolysis and aziridination reactions. Based on our assumption, the hydrolysis reaction proceeds via the protonation of the nitrene radical complex followed by the coordination of the formed hydroxy species to the iron center (Scheme 2). Therefore, we hypothesize that this reaction may be pH-sensitive and that changing the pH could be a way to control the selectivity of the overall reaction.

When examining the role of pH, it can be established that in the acidic range, at pH 4.7, the yield of the products, including the amount of SNTs (6.72%), shows a significant increase compared to the values obtained in both acetonitrile (4.16%) and the MeCN-H_2_O (pH7) buffer (1.23%). When comparing the results obtained for aqueous solutions, a significant change in the SO/SNT ratio can also be observed (5.14 at pH 7 and 3.9 at pH 4.7 buffer). At pH 8, only benzaldehyde was observed with a 5.95% yield. The λ_max_ of the 1/PhINTs adduct detectable from the reaction mixture shows a significant shift in the presence of water (and buffer at pH 4.7), which is consistent with the proposed rearrangement of the intermediate (Figure 11b).

**Figure 12:**
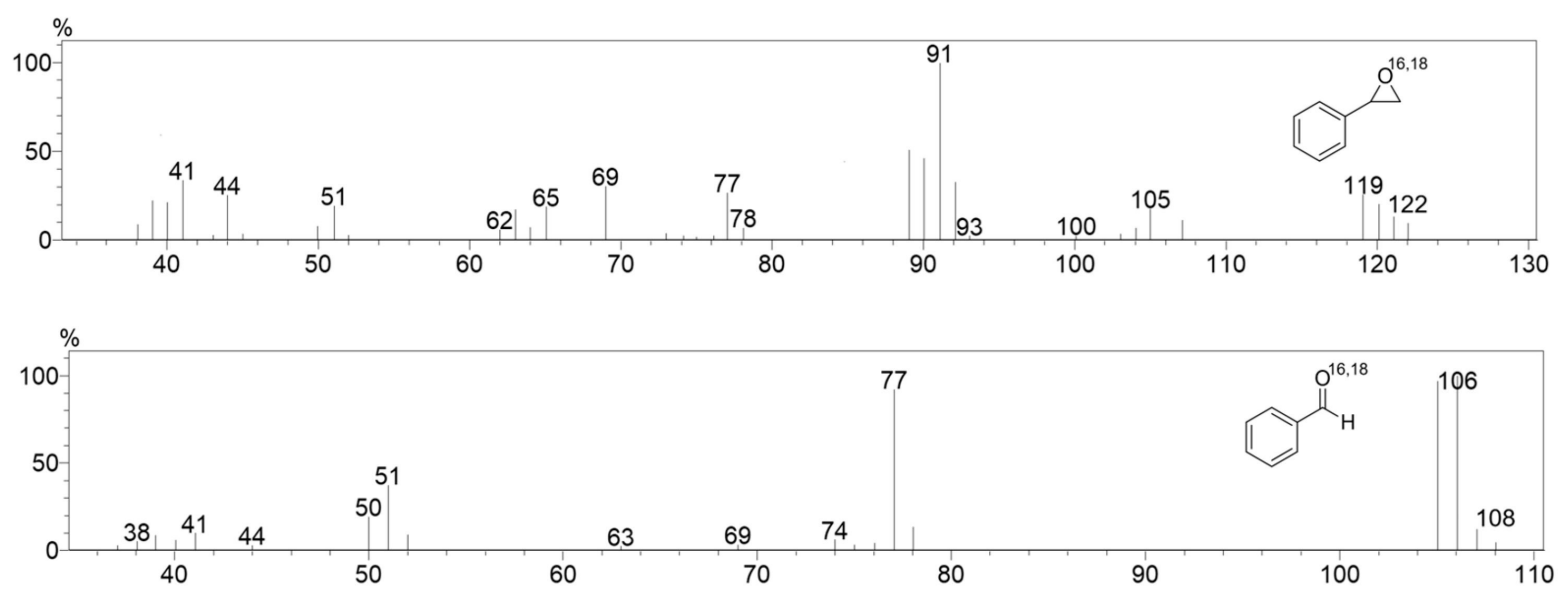
The GC-MS analysis for the catalytic oxidation of styrene in MeCN at 323 K in the presence of water, H_2_O^18^. [1]_0_ = 1 × 10^−3^ M, [PhINTs]_0_ = 1 × 10^−1^ M, [styrene]_0_ = 3 × 10^−1^ M, and [H_2_O^18^]_0_ = 1.5 × 10^−2^ M under air.

To establish the anticipated role of water in epoxide formation, the [Fe^II^(PBI)_3_(CF_3_SO_3_)_2_]-catalyzed aziridination reaction of styrene with PhINTs was reproduced in ^18^O-labeled water (Table 6). The formation of styrene oxide was confirmed with GC-MS (calcd. *m*/*z* for C_8_H_8_O: 120.0575, found *m*/*z*: 120.05). The reaction performed in ^18^O-labeled water yielded styrene oxide with an *m*/*z* of 122.05, which accurately matches styrene oxide with incorporated ^18^O (Figure 12). A similar result was observed for benzaldehyde. These results clearly demonstrate that water is the O-atom source in the formation of both styrene oxide and benzaldehyde.

In summary, styrene oxide formation during aqueous styrene aziridination most likely results from oxygen atom transfer via such in situ-formed iron oxo/oxyl radical complexes.

**Figure 13:**
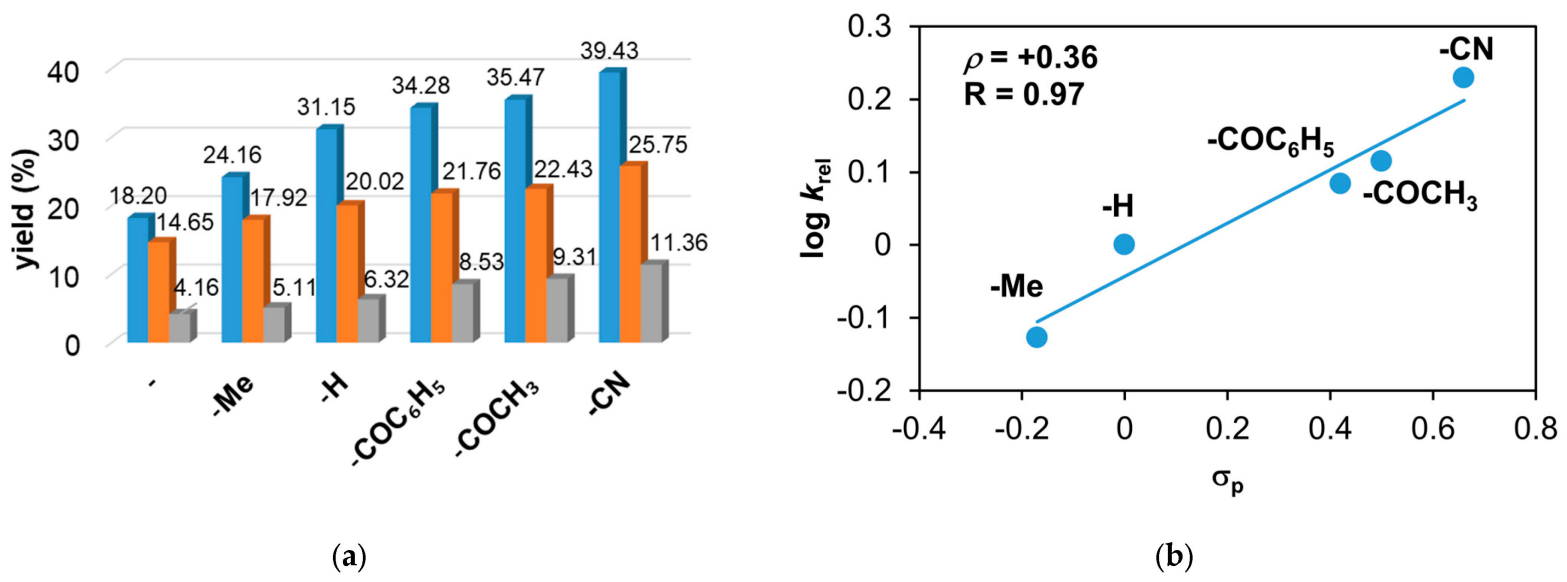
The catalytic oxidation of different *para*-substituted pyridines at 323 K in MeCN. (**a**) The yields of products for this reaction: benzaldehyde (▪), styrene oxide (▪), and 2-phenyl-1-tosylaziridine (▪). (**b**) The plot of log*k*_rel_ against σ_p_ of *para*-substituted pyridines. [1]_0_ = 1 × 10^−3^ M, [PhINTs]_0_ = 1 × 10^−1^ M, [styrene]_0_ = 3 × 10^−1^ M, and [*para*-substituted pyridine]_0_ = 1 × 10^−2^ M under air.

**Figure 14:**
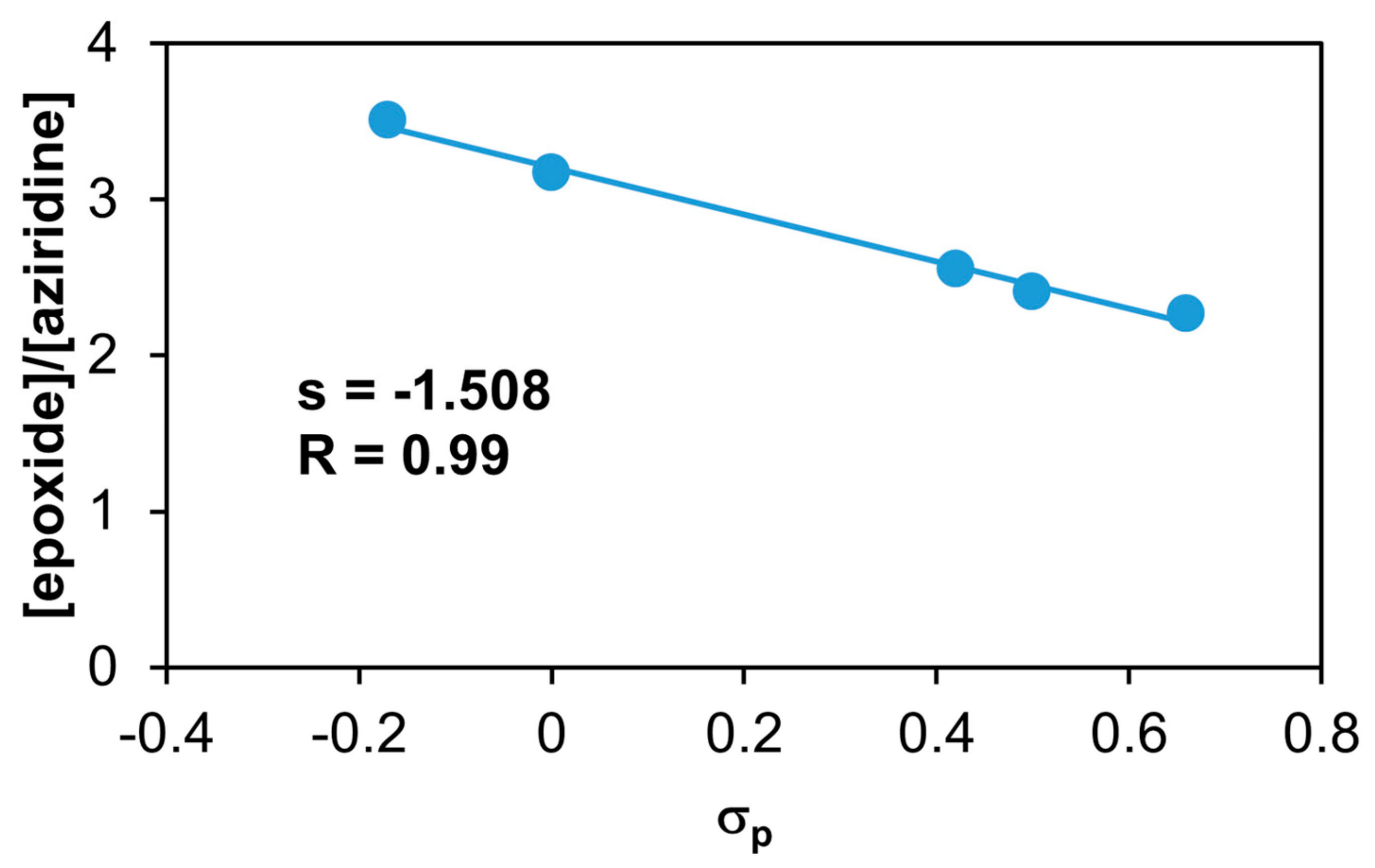
The catalytic oxidation of different *para*-substituted pyridines at 323 K in MeCN. The epoxide/aziridine ratio as a function of σ_p_. [1]_0_ = 1 × 10^−3^ M, [PhINTs]_0_ = 1 × 10^−1^ M, [styrene]_0_ = 3 × 10^−1^ M, and [*para*-substituted pyridine]_0_ = 1 × 10^−2^ M under air.

Based on our previous results, the **1**/PhIO adduct can be written with the [(PBI)_2_Fe^III^(OIPh)(Solvent)] structure [38], where the labile solvent molecules could easily be replaced by different monodentate co-ligands. Thus, their effect on electronic properties and the relationship between structure and reactivity could be investigated. Assuming a similar structure, we also examined the effect of *para*-substituted pyridine derivatives as possible co-ligands on the reactivity (selectivity) of the 1/PhINTs adduct in the aziridination reactions of styrene. Based on the obtained product yields (Figure 13a, Table 7) and the Hammett correlation (Figure 13b) that can be derived from them, it can be established that higher yields can be observed in the case of pyridine co-ligands, especially with electron-withdrawing *para*-substituents. The effect of the co-ligand on the catalyst side is smaller, but the *ρ* = +0.36 value resulting from the Hammett curve is consistent with the value obtained for the *para*-substituted styrenes (*ρ* = −1.57), indicating that the behavior of the oxidant derived from **1** and PhINTs is electrophilic. The effect of co-ligands is also reflected in the selectivity, while SO/SNTs is 3.5 for the electron-donating 4Me-Py, and it is much lower for the electron-withdrawing 4CN-Py (2.2), which is much more favorable for aziridine formation (Figure 14).

### 2.2. Stoichiometric Aziridination of Styrenes

**Figure 15:**
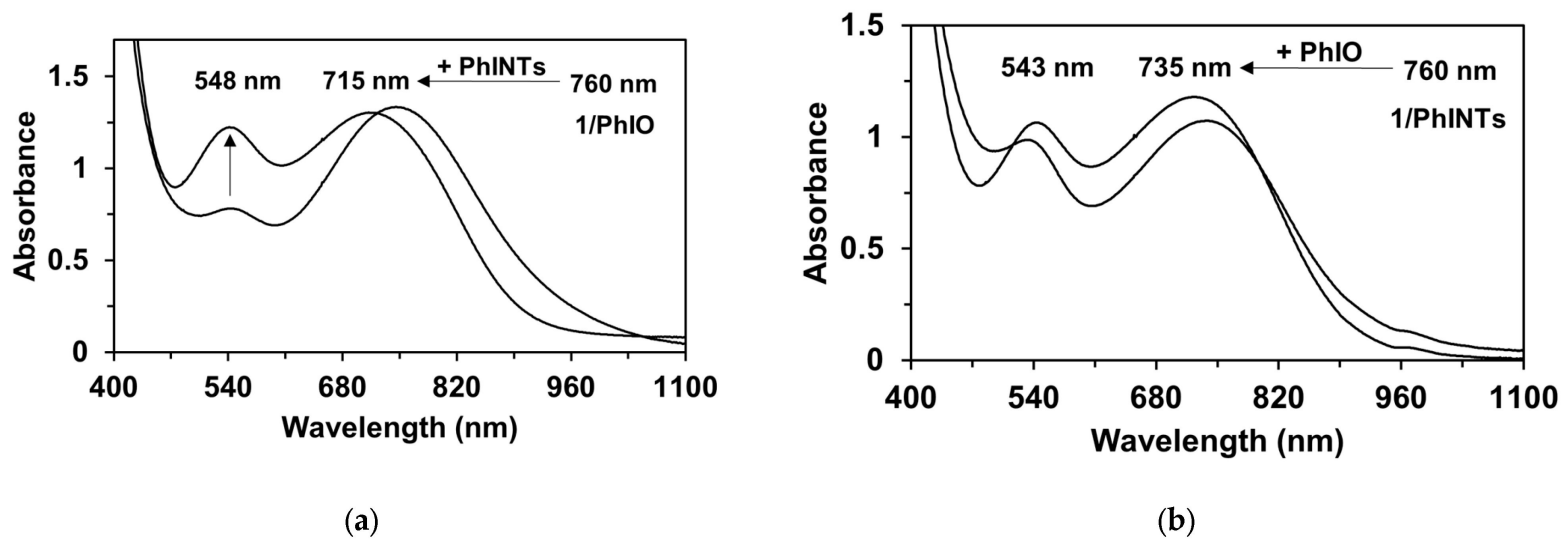
(**a**) UV-vis spectral change of Fe^III^(OIPh) with PhINTs at 293 K in MeCN. Fe^III^(OIPh) was generated in situ by reaction of **1** with PhI(OAc)_2_. (**b**) UV-vis spectral change of Fe^III^(OINTs) with PhIO at 293 K in MeCN. Fe^III^(OINTs) was generated in situ by reaction of **1** with PhINTs. [1]_0_ = 1 × 10^−3^ M, [PhINTs]_0_ = 4 × 10^−3^ M, and [PhIO]_0_ = 4 × 10^−3^ M.

Previously, to directly demonstrate the participation of the iron(III)–iodosylbenzene adduct in the epoxidation reaction, we investigated the stoichiometric reaction of Fe^III^(OIPh) produced in situ with different styrenes [38]. Similarly to the 1/PhIO adduct, the 1/PhINTs adduct generated in situ in MeCN shows a characteristic absorption around 760–770 nm. Since both epoxide and aziridine were formed in the catalytic reactions, the formation of 1/PhIO/PhINTs complexes with mixed ligands cannot be ruled out. To support this hypothesis, we investigated the reaction of the in situ-generated 1/PhIO with PhINTs (Figure 15a) and the reaction of 1/PhINTs with PhIO in acetonitrile (Figure 15b), as a result of which the λ_max_ value shifted from 760 nm to 715 nm and to 735 nm, respectively. Since the position of the band assigned to the 1/PhINTs adduct (λ_max_ = 760 nm) in acetonitrile does not change during the reaction, in this case, the formation of a 1/PhIO/PhINTs adduct is unlikely. However, similar shifts were observed during the catalytic experiments in the presence of water, and the formation of intermediates with mixed ligands in these systems cannot be ruled out (Figure 11b).

**Figure 16:**
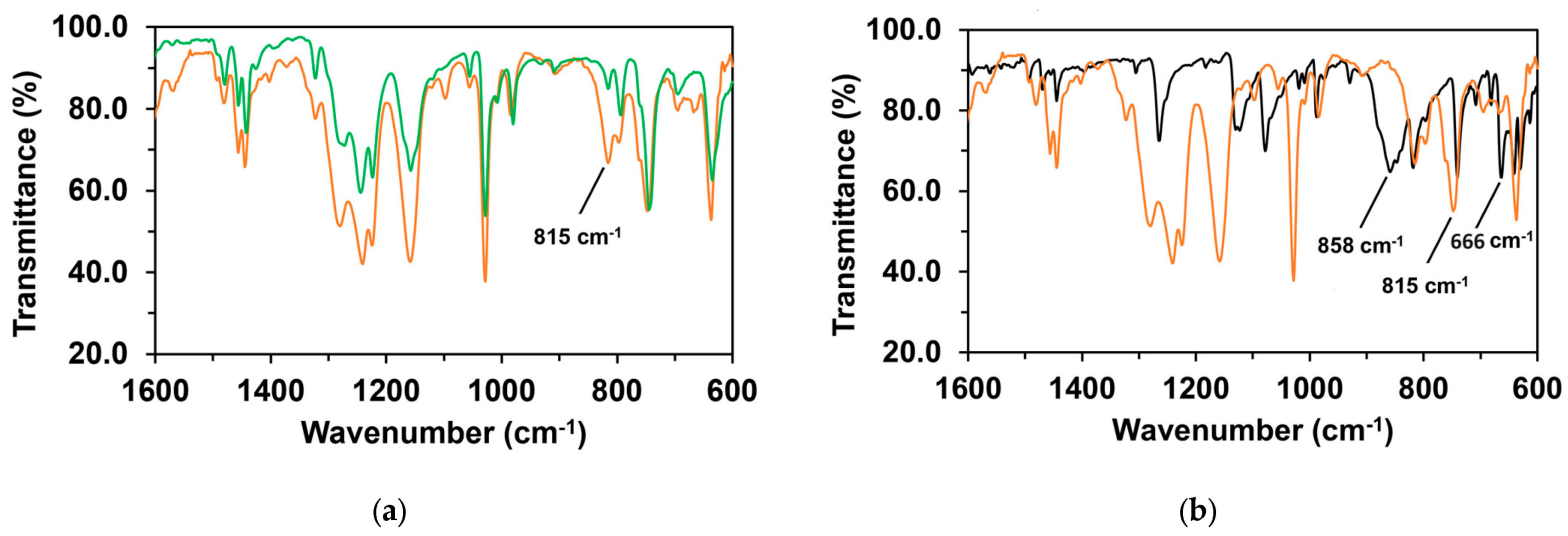
(**a**) FT-IR solid spectra of **1** (green) and **1**/PhINTs (1:1) adduct (brown). (**b**) FT-IR solid spectra of PhINTs (black) and **1**/PhINTs (1:1) adduct (brown).

In order to further prove the assumed structure of the iron adduct (Fe^IV^ = NTs/Fe^III^-N•Ts), the FT-IR spectrum of the intermediate was recorded. The infrared spectra of metal complex **1** and the **1**/PhINTs adduct show significant differences in relation to each other (Figure 16a) and to PhINTs (Figure 16b) in the range of 600–1600 cm^−1^. The peak around 815 cm^−1^ in the case of **1** can be assigned to the ν(Fe-imido) bond. The absence of the 858 and 666 cm^−1^ ν(I=N) bands, which are characteristic of PhINTs, is consistent with the splitting of PhINTs during the formation of Fe^IV^ = NTs/Fe^III^-N•Ts complexes.

**Figure 17:**
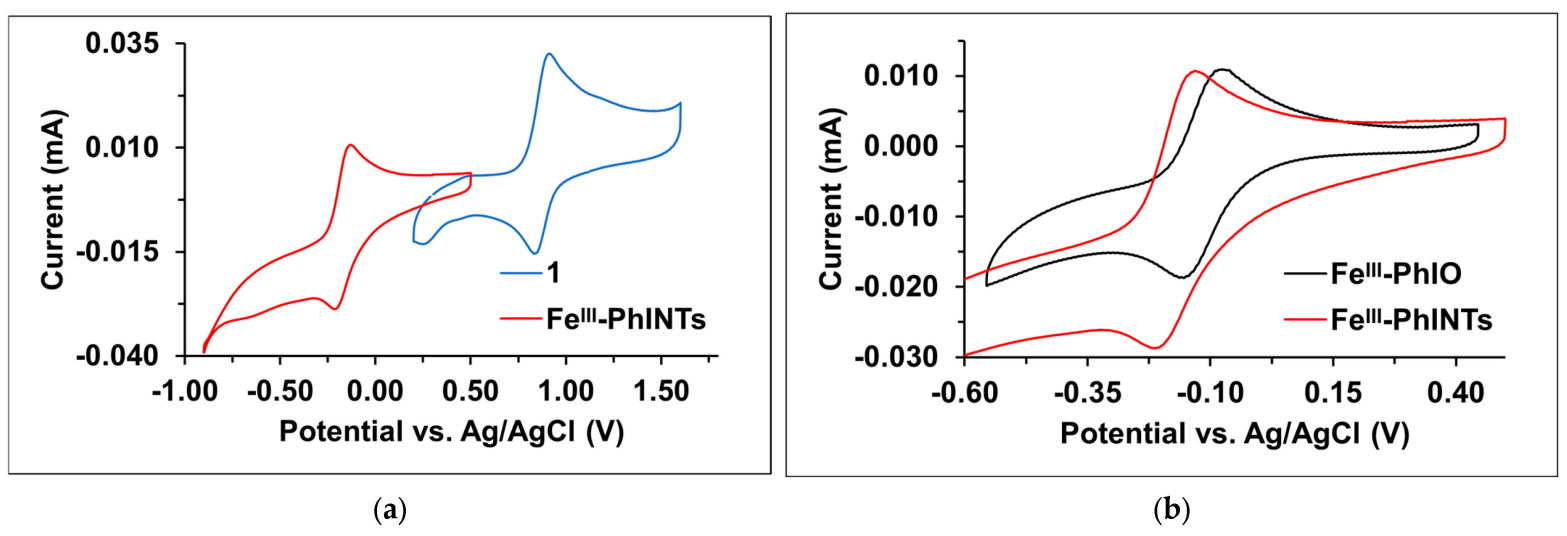
Cyclic voltammograms at 293 K in MeCN. (**a**) Cyclic voltammograms of **1** (-) and **1** with PhINTs (-). (**b**) Cyclic voltammograms of **1** with PhIO (-) and **1** with PhINTs (-). [1]_0_ = 1.0 × 10^−3^ M, PhIO = 2.0 × 10^−3^ M, and PhINTs = 2.0 × 10^−3^ M in (0.1 M TBAClO_4_) MeCN (10 cm^3^); scan rate: 500 mV/s.

The **1** complex exhibited a quasi-reversible redox couple at ca. 902 mV vs. Ag/AgCl. (Figure 17a, Table 8). Upon the addition of PhIO, there was a large change in the voltammogram, with the half-potential being found to be approximately 1017 mV more negative (Figure 17b). When PhINTs was added to the **1** complex, an even more negative half-potential was observed (−56 mV), which confirmed the electron density of the Fe^III^ d-orbitals and indicates the formation of an isoelectronic particle with the **1**/PhIO adduct (Figure 17b).

**Figure 18:**
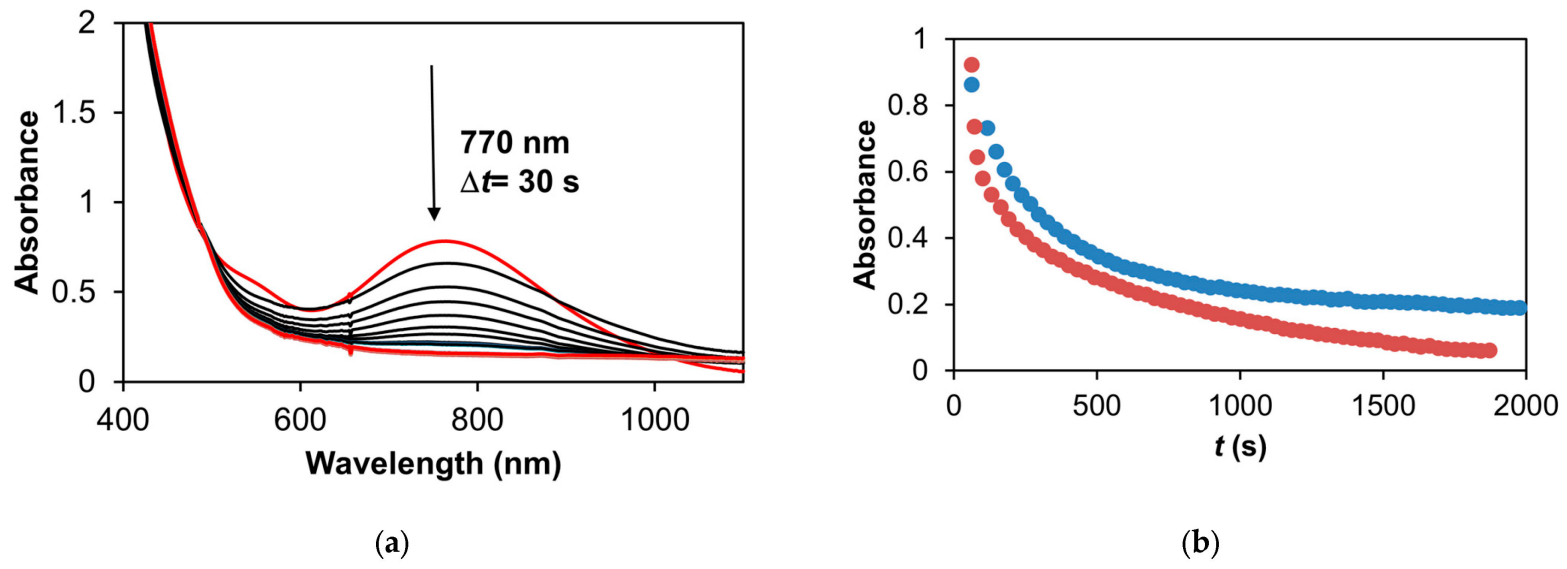
The stoichiometric oxidation of styrene with the **1**/PhINTs adduct at 293 K in MeCN. (**a**) The UV-vis spectral changes of the **1**/PhINTs adduct upon the addition of styrene. (**b**) The change in absorbance vs. *t* in the reaction of the **1**/PhINTs adduct and styrene at 770 nm in MeCN (•) and in CF_3_CH_2_OH (•). [1]_0_ = 0.5 × 10^−3^ M, [PhINTs]_0_ = 1 × 10^−3^ M, and [styrene]_0_ = 9 × 10^−1^ M.

The **1**/PhINTs adduct was prepared with **1** equivalent of PhINTs and then reacted with styrene at 293 K in acetonitrile (Figure 15a). The stoichiometric oxidation of styrene did not prove to be selective, three products were formed, and the ratio of epoxide/aziridine products was 2. The pseudo-first-order rate constants, *k*_obs_, in the reaction of **1**/PhINTs with styrenes were determined from the absorbance change at 770 nm by monitoring the decrease in the **1**/PhINTs adduct concentration (Figure 18b). An isobestic point was observed at ~525 nm, indicating that there were no long-lived intermediates during the oxidation reaction (Figure 18a).

**Figure 19:**
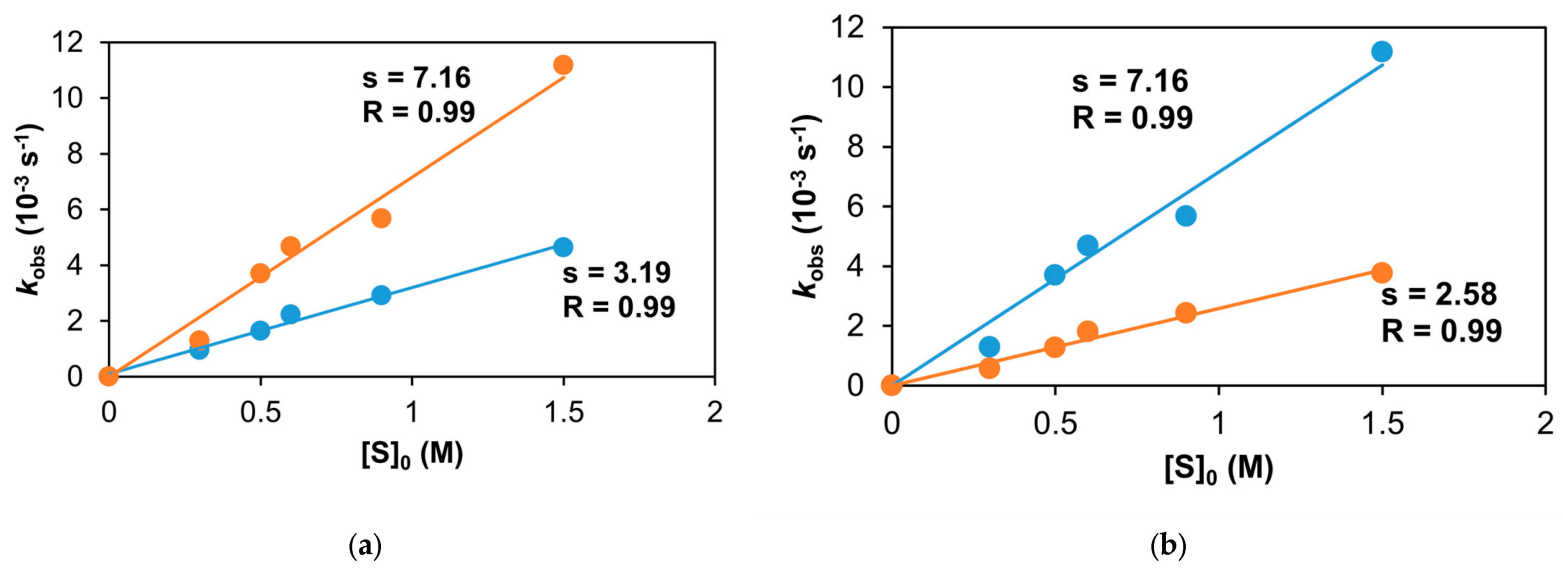
The stoichiometric oxidation of styrene at 293 K. (**a**) The reaction rate of **1**/PhIO (•) or **1**/PhINTs (•) with styrene in MeCN. (**b**) The reaction rate of the **1**/PhINTs adduct with styrene in MeCN (•) or in CF_3_CH_2_OH (•) at 293 K. [1]_0_ = 1 × 10^−3^ M; [PhINTs]_0_ = 1 × 10^−3^ M.

**Figure 20:**
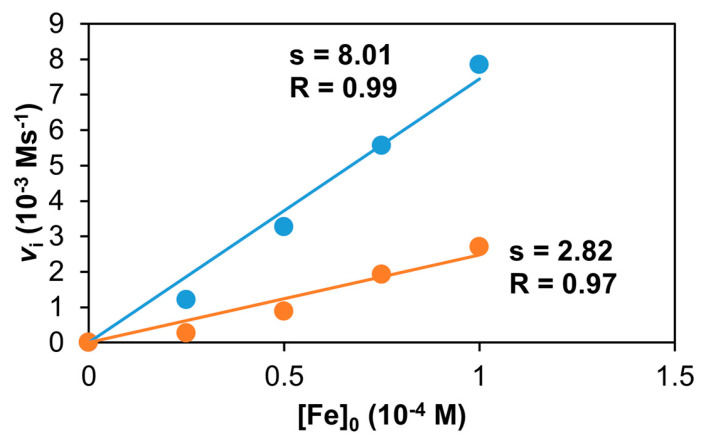
The dependence of *v_i_* on the initial complex concentration in the reaction of the **1**/PhINTs adduct and styrene in MeCN (•) or in CF_3_CH_2_OH (•) at 293 K. [styrene]_0_ = 6 × 10^−1^ M; [PhINTs]_0_ = 1 × 10^−3^ M.

In the presence of a large excess of styrene (300–1500 eq.), the rates followed pseudo-first-order kinetics (−d [**1**/PhINTs]/d*t* = *k_obs_* [**1**/PhINTs], where *k_obs_* = *k_sd_* + *k*_2_[PhCHCH_2_] and *k_sd_* << *k*_2_[PhCHCH_2_]), which can be seen as a function of the linear log (absorbance) vs. time, as well as the pseudo-first-order rate constants (*k_obs_* = *k*_2_[PhCHCH_2_)], which increased proportionally with the substrate concentration with different oxidants (Figure 19a) or in different solvents (Figure 19b). At a constant styrene concentration, the linear curve of the reaction rate values (*V*_i_ = *k_obs_* [**1**/PhINTs]) and the initial concentration of the **1**/PhINTs adduct show that the reaction is a first-order one compared to the **1**/PhINTs adduct concentration in acetonitrile and 2,2,2-trifluoroethanol (Figure 20, Table 9).

**Figure 21:**
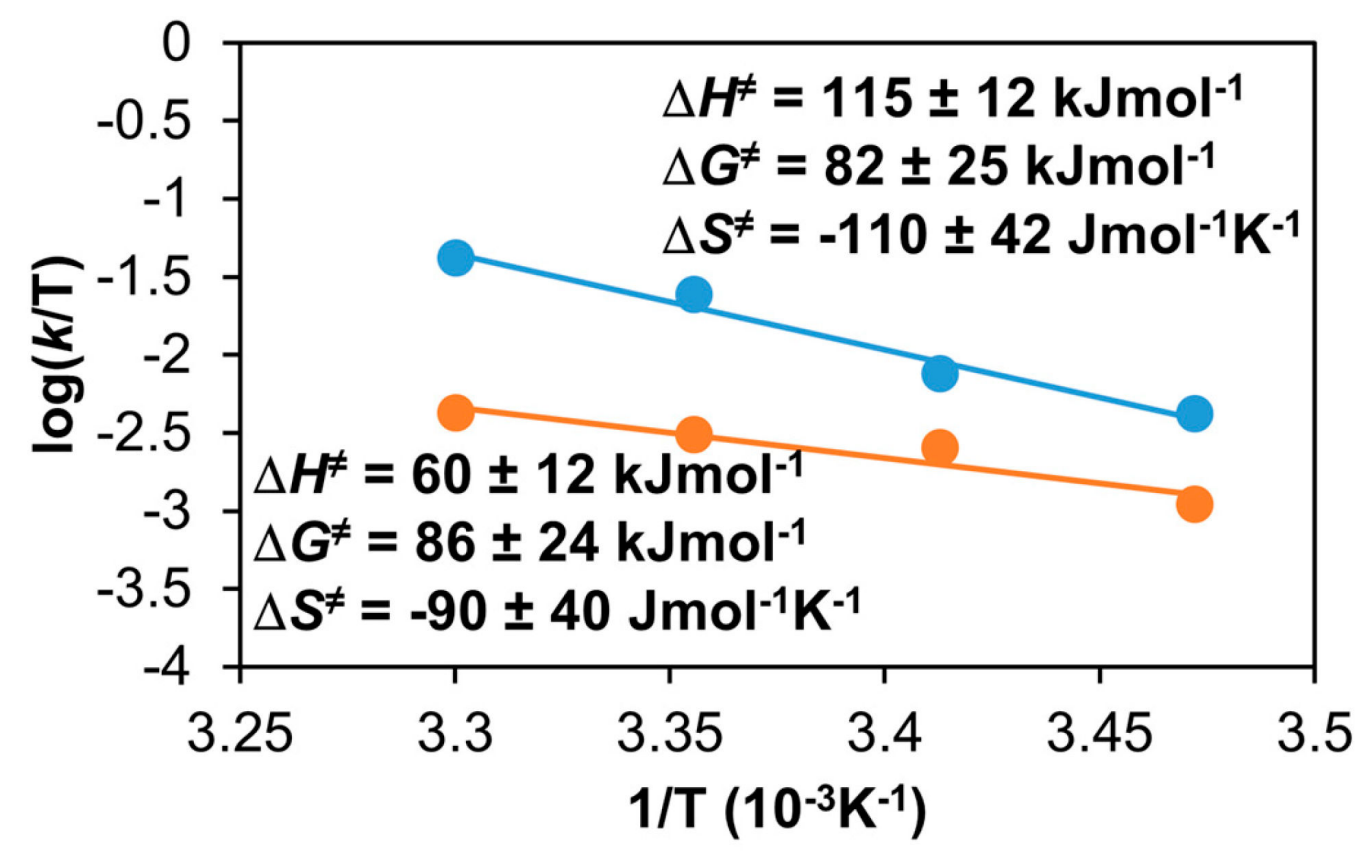
The Eyring plot of the reaction of the **1**/PhINTs adduct with styrene in MeCN (•) or in CF_3_CH_2_OH (•). [1]_0_ = 1 × 10^−3^ M, [PhINTs]_0_ = 1 × 10^−3^ M, and [styrene]_0_ = 1.5 × 10^0^ M.

Based on the above results, the following rate equation can be written for styrene derivatives; −d [**1**/PhINTs]/d*t* = *k*_2_[**1**/PhINTs][PhCHCH_2_]. From the performed experiments, the second-order rate constant (*k*_2_) was determined as 7.16 × 10^−3^ M^−1^ s^−1^, where Δ*H*^≠^ = 115 ± 12 kJ mol^−1^, Δ*S*^≠^ = −110 ± 42 J mol^−1^ K^−1^, Δ*G*^≠^ = 82 ± 25 kJ mol^−1^ at a temperature of 293 K in acetonitrile, and 2.58 × 10^−3^ M^−1^ s^−1^, where Δ*H*^≠^ = 60 ± 12 kJ mol^−1^, Δ*S*^≠^ = −90 ± 40 J mol^−1^ K^−1^, and Δ*G*^≠^ = 86 ± 24 kJ mol^−1^ at a temperature of 293 K in 2,2,2-trifluoroethanol, respectively (Figure 21). These values are much higher than what was established for Fe^IV^(O) species containing pentadentate polypyridyl ligands (*k*_2_ = 2.94 × 10^−4^ M^−1^ s^−1^, Δ*G*^≠^ = 93 ± 8 kJ mol^−1^ 298 K) [42]. Comparing the obtained values, it can be concluded that the oxidation of styrene in acetonitrile is more favorable than in 2,2,2-trifluoroethanol, and the reaction with PhINTs takes place at a much higher rate than with PhIO. The lower Δ*G*^≠^ value obtained for PhINTs in acetonitrile is also congruous with the above result. These results also agree with those observed in catalysis, according to which higher yields were achieved in acetonitrile. The relatively large, negative entropies are characteristic of associative processes.

**Figure 22:**
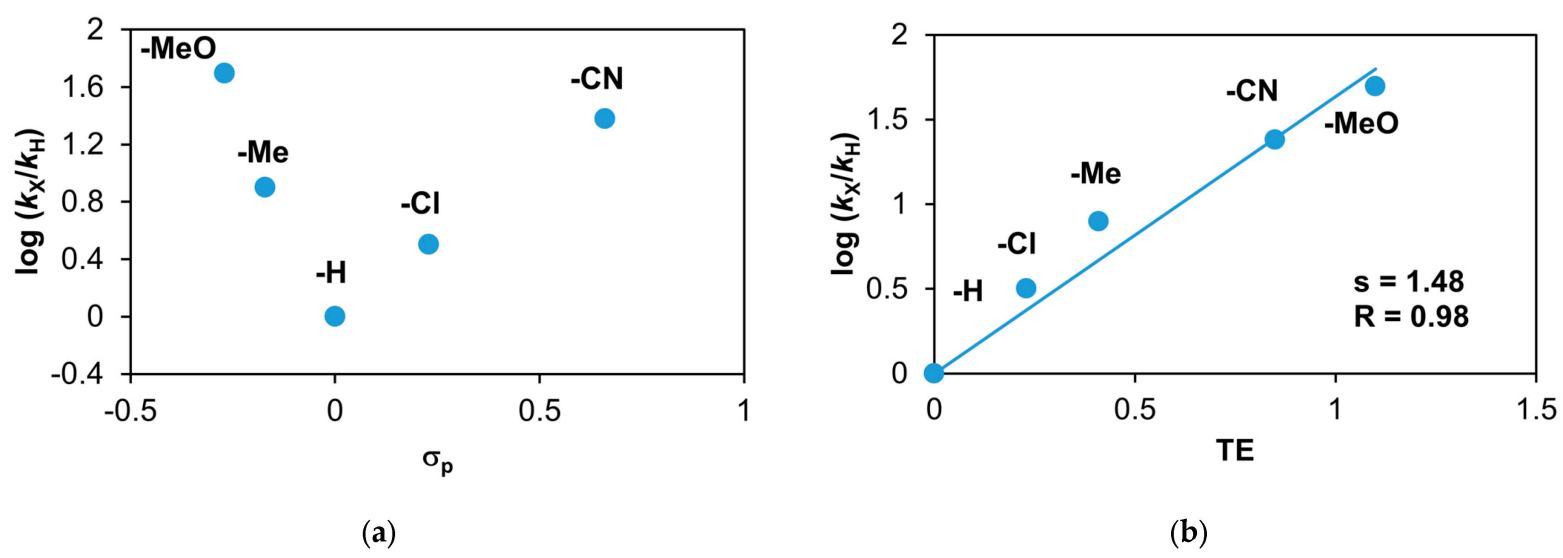
Stoichiometric oxidation of different *para*-substituted styrenes at 293 K in MeCN. (**a**) Plot of log(*k*_X_/*k*_H_) against σ_p_ of *para*-substituted styrenes. (**b**) Plot of log(*k*_X_/*k*_H_) against TE of *para*-substituted styrenes. [1]_0_ = 1 × 10^−3^ M, [PhINTs]_0_ = 1 × 10^−3^ M, and [styrenes]_0_ = 5 × 10^−1^ M.

**Figure 23:**
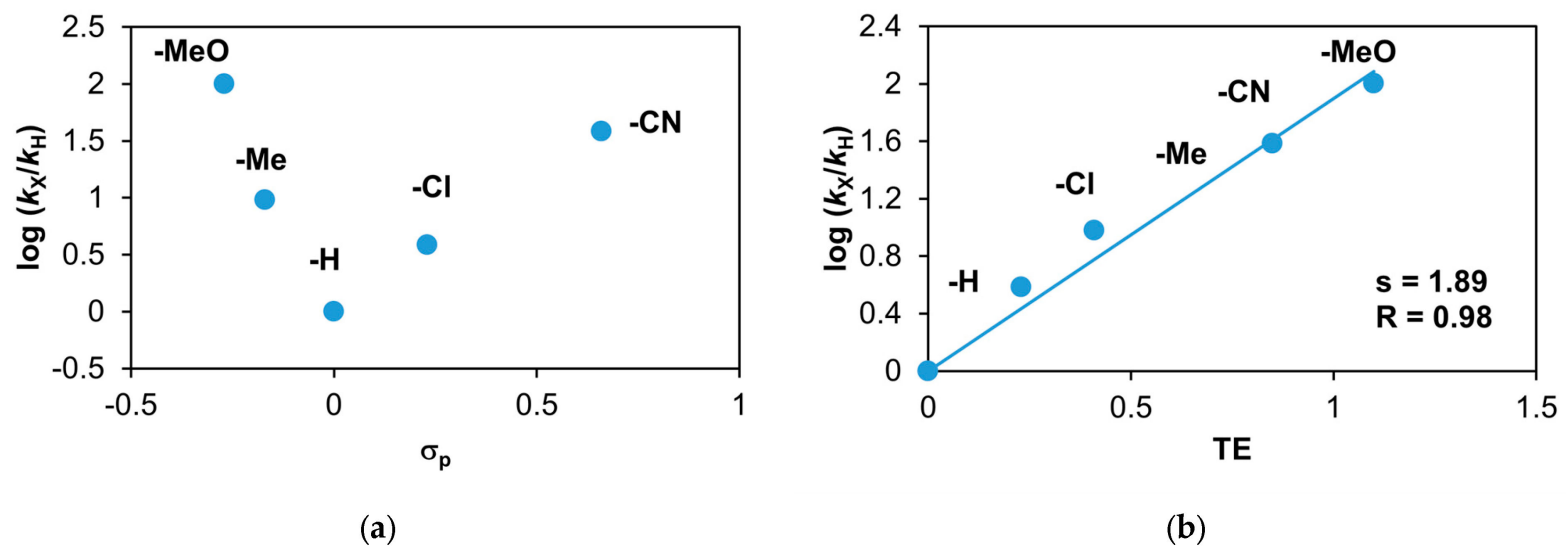
Stoichiometric oxidation of different *para*-substituted styrenes at 293 K in CF_3_CH_2_OH. (**a**) Plot of log(*k*_X_/*k*_H_) against σ_p_ of *para*-substituted styrenes. (**b**) Plot of log(*k*_X_/*k*_H_) against TE of *para*-substituted styrenes. [1]_0_ = 1 × 10^−3^ M, [PhINTs]_0_ = 1 × 10^−3^ M, and [styrenes]_0_ = 5 × 10^−1^ M.

The studies were extended to *para*-substituted styrenes to assess the effect of electronic factors on the reaction. Electron-sending and electron-withdrawing substituents both speed up the reaction, so plotting the relative reactivity values (log *k*_rel_) as a function of the substituent constants (*σ*_p_) of the *para*-substituted styrenes leads to a concave Hammett curve rather than a straight line in acetonitrile and in 2,2,2-trifluoroethanol (Figure 22a and Figure 23a; Table 10) [43]. The obtained Hammett relationship apparently contradicts the results obtained under catalytic conditions, which can be explained by the fact that, in addition to the large excess of oxidant, other side reactions also appear. Plotting the relative rate of the oxidation of *para*-substituted styrenes (log*k*_rel_) as a function of the total substituent effect (TE, stabilities of the benzylic radicals including spin delocalization and polar effects) resulted in a linear free energy relationship, where *ρ*TE• = +1.48 (in acetonitrile) and +1.89 (in 2,2,2-trifluoroethanol) (Figure 22b and Figure 23; Table 10).

## 3. Materials and Methods

All chemicals, including the PBI ligand and styrenes, were obtained from Aldrich Chemical Co. (Milwaukee, WI, USA) and used without further purification unless otherwise noted. Solvents were dried according to published procedures and distilled, stored under argon [44]. [Fe^II^(PBI)_3_](OTf)_2_ and PhINTs were produced by literature methods [45,46]. The UV-visible spectra were recorded on an Agilent 8453 diode-array spectrophotometer using quartz cells. IR spectra were recorded using an Shimadzu IR Affinity-1s spectrophotometer (SHIMKON Kft, Budapest, Hungary), with a MIRacle 10 (Diamon/ZnSe) one reflection ATR plate, using the LabSolutions IR Series Software (A22135801102). GC analysis was performed on an Agilent 6850 (Budapest, Hungary) gas chromatograph equipped with a flame ionization detector with a 30 m HP-5 column. GC-MS analyses were performed on a Shimadzu QP2010SE (Budapest, Hungary) equipped with a secondary electron multiplier detector with conversion and a 30 m HP5-MS column. Cyclic voltammetric experiments were carried out using an SP-150 potentiostat, using the EC-Lab V11.41 software (Labronite, Debrecen, Hungary).

### 3.1. Cyclic Voltammetry

During the measurements, we used a three-electrode setup, we used a 3.0 mm diameter glassy carbon electrode as working electrode, a Pt wire as counter electrode, and an Ag/AgCl (3 M KCl) reference electrode. The supporting electrolyte was a 0.1 M solution of tetrabutylammoniumperchlorate.

### 3.2. Oxidation Conditions

#### 3.2.1. Stoichiometric Oxidation

[Fe^II^(PBI)_3_](OTf)_2_ (1 × 10^−3^ M, 1.88 mg) was dissolved in acetonitrile (2 mL) or 2,2,2-trifluoroethanol and PhINTs (1 × 10^−3^ M, 0.75 mg) was added to the solution. The mixture was stirred until the intermediate was formed, and then the substrate (0.3–1.5 M, 69–345 μL) was added to the solution and the reaction was monitored with UV-vis spectrophotometer at 770 nm. The product was identified by GC-MS, and the yields were determined by GC using bromobenzene as internal standard. FT-IR (solid) data for 1/PhINTs adduct are as follows: 1605, 1456, 1445, 1279, 1240, 1225, 1157, 1028, 984, 816, 796, 636 cm^−1^.

#### 3.2.2. Catalytic Oxidation

[Fe^II^(PBI)_3_](OTf)_2_ (1 × 10^−3^ M, 2.82 mg) was dissolved in acetonitrile or 2,2,2-trifluoroethanol (3 mL) under air (argon) atmosphere, PhINTs (1 × 10^−1^ M, 112 mg) and substrate (3 × 10^−1^ M, 103 μL), and, in some cases, water (1.5 × 10^−2^ M–2× 10^−1^ M), D_2_O (1.5 × 10^−2^ M), and buffer (200 μL) were added to the solution. Bromobenzene (1× 10^−1^ M, 31 μL) was added to the solution as an internal standard. The mixture was stirred for 1–4 h at 323 K, and then the products were identified by GC-MS and the yields were determined by GC.

#### 3.2.3. The Composition of the Designated Buffer

pH 4.7: 10 mmol (2.87 mL) glacial acetic acid and 10 mmol (6.8 g) sodium acetate trihydrate were dissolved in 200 mL water in a volumetric flask. The buffer was adjusted with KOH and HCl.

pH 8: 6.25 mmol (2.38 g) borax was dissolved in 250 mL water in a volumetric flask, 25 mmol (2.09 mL) HCl (37% m/m) was dissolved in 250 mL water in a volumetric flask, and 41.0 mL HCl solution was added to 100 mL borax solution. The buffer was adjusted with KOH and HCl.

### 3.3. Characterization of Oxidation Products

#### 3.3.1. Products of Styrene Oxidation

Benzaldehyde: (*m*/*z*) relative intensity 106.00 (M^+^, 86.93); 105.00 (85.30); 78.00 (23.29); 77.00 (100); 76.05 (7.11); 74.00 (14.76); 52.00 (17.78); 51.00 (69.04); 50.00 (41.93); 49.00 (5.26); 39.00 (12.45); 38.05 (8.64); 37.05 (6.04).

Styrene oxide: (*m*/*z*) relative intensity 120.00 (M^+^, 23.12); 119.05 (41.61); 92.05 (39.66); 91.05 (100); 90.05 (62.92); 89.00 (91.83); 77.00 (14.60); 73.95 (6.17); 65.05 (28.03); 64.00 (13.69); 63.00 (36.01); 62.00 (14.33); 51.00 (32.55); 50.00 (18.21); 39.00 (35.28); 38 (13.03).

2-Phenyl-1-tosylaziridine: (*m*/*z*) relative intensity 273.1 (M^+^, 11.05); 139.00 (12.09); 118.05 (53.59); 117.05 (6.75); 91.00 (100); 89.05 (10.32); 65.00 (26.64); 63.00 (6.81); 39.00 (9.44).

#### 3.3.2. H_2_O^18^ Labeling Experiment

Benzaldehyde: *m*/*z* (%) = 106 (100) [M^+^], 105 (98.3), 77 (90.8), 51 (39.6); 108 (4.9) [M^+^ + 2], 106 (100), 105 (97.1), 77 (92.4), 51 (37.5).

Styrene oxide: *m*/*z* (%) = 120 (27.6) [M^+^], 119 (35.3), 104 (4.9), 92 (32.3), 91 (100), 90 (40.5), 89 (46.3), 77 (7.1); 122 (13.1) [M^+^ + 2], 121 (13.6), 120 (20.9), 119 (27.3), 105 (14.8), 104 (6.8), 92 (34.8), 91 (100), 90 (48), 89 (51.1), 77 (23.8).

#### 3.3.3. Products of 4-Vinylanisole Oxidation

4-Methoxybenzaldehyde: (*m*/*z*) relative intensity 136.05 (M^+^, 70.83); 135.05 (100); 107.00 (20.24); 92.00 (28.23); 78.00 (8.74); 77.00 (56.32); 76.05 (6.89); 74.05 (7.70); 65.05 (24.99); 64.00 (22.60); 63.00 (28.21); 62.00 (8.67); 51.00 (18.87); 50.00 (18.39); 39.05 (32.99); 38.05 (13.55).

2-(4-methoxyphenyl)oxirane: (*m*/*z*) relative intensity 150.05 (M^+^, 36.75); 135.00 (72.44); 119.00 (55.09); 92.05 (39.45); 91.05 (100); 90.00 (57.75); 89.00 (64.67); 77.00 (29.84); 74.05 (16.43); 65.00 (35.32); 64.05 (16.21); 63.00 (34.82); 62.05 (8.53); 51.05 (25.05); 50.00 (22.57); 41.00 (17.15); 39.05 (32.75); 38.00 (15.36).

2-(4-methoxyphenyl)-1-tosylaziridine: (*m*/*z*) relative intensity 303.09 (M^+^, 58.14); 273.05 (12.17); 139.05 (15.16); 118.00 (51.39); 117.00 (8.17); 91.05 (100); 89.05 (14.62); 65.05 (23.15); 63.05 (8.16); 39.05 (8.16).

#### 3.3.4. Products of 4-Methylstyrene Oxidation

4-Methylbenzaldehyde: (*m*/*z*) relative intensity 121.05 (5.72); 120.00 (M^+^; 62.78); 119.00 (68.19); 92.05 (10.63); 91.05 (100); 89.05 (13.96); 65.05 (45.16); 63.05 (23.50); 62.00 (11.01); 50.95 (14.50); 50.05 (12.28); 41.10 (5.17); 39.15 (35.78); 38.05 (7.59).

2-(4-methylphenyl)oxirane: (*m*/*z*) relative intensity 134.05 (M^+^, 32.51); 119.00 (100); 92.05 (49.15); 91.00 (66.16); 90.00 (59.42); 89.00 (67.14); 77.05 (29.15); 74.00 (15.42); 65.05 (27.18); 64.00 (19.93); 63.00 (40.64); 62.05 (18.16); 51.05 (26.15); 50.00 (37.24); 41.00 (16.93); 39.00 (38.71); 38.05 (14.74).

2-(p-tolyl)-1-tosylaziridine: (*m*/*z*) relative intensity 288.05 (M^+^, 18.36); 287.00 (73.71); 273.10 (100); 139.05 (16.16); 118.05 (57.52); 117.00 (8.17); 91.00 (45.74); 89.00 (8.73); 65.05 (29.38); 63.05 (9.16); 39.05 (11.45).

#### 3.3.5. Products of 4-Chlorostyrene Oxidation

4-chlorobenzaldehyde: (*m*/*z*) relative intensity 141.95 (23.67); 141.00 (37.15); 140.00 (M^+^, 63.53); 139.00 (100); 113.00 (20.62); 112.00 (15.72); 111.00 (66.23); 85.00 (9.78); 84.05 (6.35); 77.00 (40.48); 76.00 (21.71); 75.05 (77.71); 74.00 (49.35); 73.00 (14.02); 62.95 (5.18); 61.95 (7.75); 61.00 (9.55); 51.00 (51.34); 50.00 (81.80); 49.05 (14.91); 39.05 (7.35); 38.00 (14.65); 37.10 (16.39).

2-(4-chlorophenyl)oxirane: (*m*/*z*) relative intensity 153.90 (M^+^, 26.15); 127.05 (12.05); 126.05 (6.69); 125.05 (45.34); 124.15 (14.00); 119.10 (36.23); 99.05 (8.98); 91.05 (29.95); 90.05 (12.57); 89 (100); 86.95 (6.79); 77.00 (5.43); 75.00 (9.60); 74.05 (6.70); 72.90 (7.80); 63 (31.56); 61.95 (14.95); 60.90 (8.10); 50.95 (15.35); 49.90 (19.61); 39.05 (15.59); 38.05 (7.20).

2-(4-chlorophenyl)-1-tosylaziridine: (*m*/*z*) relative intensity 309.05 (36.18); 307.05 (M^+^, 100); 271.10 (19.25); 139.05 (13.52); 118.00 (58.16); 117.05 (5.22); 91.00 (78.15); 89.05 (9.15); 65.00 (28.09); 63.00 (6.84); 39.00 (22.97).

#### 3.3.6. Products of 4-Cyanostyrene Oxidation

4-cianobenzaldehyde: (*m*/*z*) relative intensity 131.00 (M^+^, 64.57); 130.00 (100); 103.00 (29.72); 102.00 (77.29); 77.00 (8.34); 76.00 (57.07); 75.00 (56.22); 74.00 (23.50); 73.00 (7.39); 63.00 (8.24); 62.00 (6.64); 61.00 (7.44); 52.05 (13.90); 51.00 (42.61); 50.00 (54.77); 49.00 (10.55); 39.05 (12.2); 38.00 (13.43); 37.05 (13.34).

2-(4-cianophenyl)oxirane: (*m*/*z*) relative intensity 145.10 (M^+^, 43.26); 119.05 (100); 91.95 (41.15); 91 (46.17); 90.05 (48.91); 89.00 (50.63); 77.00 (32.53); 74.00 (18.16); 65.00 (37.63); 64.00 (15.04); 63.00 (36.88); 62.00 (14.24); 51.05 (45.14); 50.00 (37.78); 41.00 (21.63); 39.00 (39.72); 38.00 (20.13).

4-(1-tosylaziridin-2-yl)benzonitrile: (*m*/*z*) relative intensity 299.08 (M^+^, 17.45); 298.08 (100); 273.1 (13.05); 139 (10.16); 118.05 (55.27); 117.00 (7.15); 91.05 (88.23); 89.00 (8.28); 65.05 (23.37); 63.05 (7.54); 39.05 (11.22).

#### 3.3.7. Products of α-Methylstyrene Oxidation

Acetophenone: (*m*/*z*) relative intensity 120.10 (M^+^, 18.19); 105.20 (42.38); 76.90 (100); 75.95 (15.67); 74.90 (8.66); 73.90 (14.50); 73.05 (9.50); 63.10 (15.77); 62.05 (14.48); 60.80 (14.47); 51.00 (84.48); 50.00 (43.33); 44.05 (13.04); 43.10 (41.74); 42.10 (14.85); 40.70 (5.79); 38.95 (16.50); 38.00 (24.21); 37.05 (5.39).

2-methyl-2-phenyloxirane: (*m*/*z*) relative intensity 134.20 (M^+^, 16.24); 133.20 (26.23); 105.15 (100); 104.10 (25.44); 103.05 (39.18); 91.05 (18.04); 88.90 (5.24); 79.90 (8.47); 78.95 (14.74); 78.10 (61.99); 76.90 (46.55); 74.80 (6.31); 73.85 (9.46); 65.15 (17.26); 64.00 (9.86); 63.05 (22.47); 62.05 (27.86); 53.20 (12.57); 50.90 (57.49); 49.95 (25.62).

2-methyl-2-phenyl-1-tosylaziridine: (*m*/*z*) relative intensity 287.10 (M^+^, 38.42); 272.05 (15.43); 196.00 (25.75); 132.10 (26.93); 105.10 (100); 104.05 (29.34); 103.15 (26.48); 91.10 (14.04); 89.05 (7.18); 80.05 (6.75); 79.05 (19.41); 78.10 (49.28); 77.05 (48.15); 75.00 (8.15); 74.05 (10.54); 65.05 (22.83); 64.05 (7.62); 63.10 (26.93); 62.10 (23.06); 53.10 (17.53); 51.00 (53.86); 50.00 (27.25).

## 4. Conclusions

In summary, we investigated the aziridination reaction of various *para*-substituted styrene derivatives (both catalytic and stoichiometric) using a nonheme [Fe^II^(PBI)_3_](OTf)_2_ (**1**) complex as a catalyst and PhINTs as an oxidizing agent in the presence and absence of pyridine additives. We found that the average conversion values achieved in acetonitrile during catalysis decreased in protic 2,2,2-trifluoroethanol, while styrene containing electron-donating substituents increased conversion in both solvents. In the case of pyridine additives, the highest increase in reactivity was achieved in the case of pyridines containing electron-withdrawing substituents. Based on the Hammett results for the substrate and co-ligand above, an electrophilic 1/PhINTs adduct as a reactive intermediate can be proposed. The involvement of the nitrene radical complex Fe^III^(N•Ts) is supported by the absence of product formation when the 2,6-ditertbutyl-4-methylphenol as a radical scavenger was present in the reaction mixture. Based on the linear free energy relationships between the relative reaction rates and total substituent effect (TE) parameters with slopes of 1.48 (MeCN) and 1.89 (CF_3_CH_2_OH), a nonconcerted electron transfer (ET) mechanism through the formation of the radicaloid benzylic radical intermediate can be proposed for stoichiometric aziridination reactions. We also found that the aziridination reactions in all cases yielded oxygen-containing epoxides and aldehydes next to the desired aziridine products, which can be explained by the presence of trace water in the organic solvents. The observed SIE from the H_2_O- and D_2_O-containing experiments clearly confirmed the role of water as the oxygen source. Namely, the hydrolysis of the formed nitrene radical complexes yields Fe^IV^(O)/Fe^III^O• radical complexes active in oxygen atom transfer catalysis (Scheme 2). Overall, we demonstrated that understanding the reactivity and selectivity of nitrene (radical) complexes in the presence of water is fundamental to control and improve aqueous (radical-type) nitrene transfer catalysis.

## Figures and Tables

**Table 1:** The yields of products (benzaldehyde, styrene oxide, and 2-phenyl-1-tosylaziridine) for the catalytic oxidation of styrene at 323 K in MeCN at different times. [1]_0_ = 1 × 10^−3^ M, [PhINTs]_0_ = 1 × 10^−1^ M, and [styrene]_0_ = 3 × 10^−1^ M under air.

Entry	t (min)	Yield (%) ^1^			Selectivity (%)			TON ^2^	TOF (1/h) ^3^
		BZ	SO	SNTs	BZ	SO	SNTs		
1	60	6.218	3.98	0.8	57	36	7	10.99	2.7495
2	120	11.92	6.78	1.4	59	34	7	20.1	5.025
3	180	15.64	11.32	2.43	53	39	8	29.39	7.3475
4	240	18.2	14.65	4.16	49	40	11	37.01	9.2525
5	300	18.4	14.95	4.19	49	40	11	37.54	9.385

^1^ Based on PhNTs, whose values are identical with the efficiency values. ^2^ TON = [Substrate]_0_/[1]_0_. ^3^ TOF = TON/h.

**Table 2:** The yields and selectivity of products (Bz, benzaldehyde; SO, styrene oxide; and SNTs, 2-phenyl-1-tosylaziridine) for the catalytic oxidation of styrene at different temperatures in MeCN. [1]_0_ = 1 × 10^−3^ M, [PhINTs]_0_ = 1 × 10^−1^ M, and [styrene]_0_ = 3 × 10^−1^ M under air.

Entry	T (K)	Yield (%) ^1^			Selectivity (%)			TON ^2^	TOF (1/h) ^3^
		BZ	SO	SNTs	BZ	SO	SNTs		
1	293	16.01	9.24	2.15	58	34	8	27.4	6.85
2	308	17.02	10.52	4.41	53	33	14	31.95	7.99
3	323	18.2	14.65	4.16	49	45	11	37.01	9.25
4	338	6.41	4.8	1.52	50	38	12	12.73	3.18

^1^ Based on PhNTs, whose values are identical with the efficiency values. ^2^ TON = [Substrate]_0_/[1]_0_. ^3^ TOF = TON/h.

**Table 3:** The yields and selectivity of products (benzaldehyde, styrene oxide, and 2-phenyl-1-tosylaziridine) for the catalytic oxidation of styrene in MeCN at 323 K. [PhINTs]_0_ = 1 × 10^−1^ M; [styrene]_0_ = 3 × 10^−1^ M under air.

Entry	[1]_0_ (10^−3^ M)	Yield (%) ^1^			Selectivity (%)			TON ^2^	TOF (1/h) ^3^
		BZ	SO	SNTs	BZ	SO	SNTs		
1	0.5	3.17	2.13	1.19	49	33	18	6.49	1.62
2	1	18.2	14.65	4.16	49	40	11	37.01	9.25
3	2	19.25	20.23	6.34	42	44	14	45.82	11.45

^1^ Based on PhNTs, whose values are identical with the efficiency values. ^2^ TON = [Substrate]_0_/[1]_0_. ^3^ TOF = TON/h.

**Table 4:** The yields of products (Bz, benzaldehyde; SO, styrene oxide; and SNTs, 2-phenyl-1-tosylaziridine) and calculated TON and TOF values for the catalytic oxidation of *para*-substituted styrenes at 323 K in MeCN. [1]_0_ = 1 × 10^−3^ M, [PhINTs]_0_ = 1 × 10^−1^ M, and [styrene]_0_ = 3 × 10^−1^ M under air.

Entry	Substrate	Yield (%) ^3^			Selectivity (%)			TON ^4^	TOF (1/h) ^5^
	4R-S	BZ	SO	SNTs	BZ	SO	SNTs		
1	4-methoxystyrene	32.35	20.21	10	52	32	16	62.56	15.64
2	4-methylstyrene	22	17.34	8.51	46	36	18	47.85	11.96
3	styrene	18.2	14.65	4.16	49	40	11	37.01	9.25
4	styrene ^1^	-	-	-	-	-	-	-	-
5	4-chlorostyrene	6.34	4.94	1.27	51	39	10	12.55	3.13
6	4-cyanostyrene	1.4	0.77	0.21	59	32	9	2.38	0.59
7	α-methylstyrene	3.19 ^2^	5.16	0.61	35	58	7	8.96	2.24
8	styrene-d_8_	6.4	4.9	1.4	50	39	11	12.7	3.17

^1^ 5 × 10^−3^ M 2,6-ditertbutyl-4-methylphenol. ^2^ Acetophenone. ^3^ Based on PhNTs, whose values are identical to the efficiency values. ^4^ TON = [Substrate]_0_/[1]_0_. ^5^ TOF = TON/h under air.

**Table 5:** The yields of products (benzaldehyde, styrene oxide, and 2-phenyl-1-tosylaziridine) and calculated TON and TOF values for the catalytic oxidation of *para*-substituted styrenes at 323 K in CF_3_CH_2_OH. [1]_0_ = 1 × 10^−3^ M, [PhINTs]_0_ = 1 × 10^−1^ M, and [styrene]_0_ = 3 × 10^−1^ M under air.

Entry	Substrate	Yield (%) ^1^			Selectivity (%)			TON ^2^	TOF (1/h) ^3^
	4R-S	BZ	SO	SNTs	BZ	SO	SNTs		
1	4-methoxystyrene	17.7	7.89	1.3	66	29	5	26.89	6.72
2	4-methylstyrene	9.3	4.71	0.47	65	32	3	14.48	3.62
3	styrene	3.88	2.67	1.87	46	32	22	8.42	2.11
4	4-chlorostyrene	2.05	0.36	0.38	73	13	14	2.79	0.69
5	4-cyanostyrene	0.48	0.27	0.26	48	27	25	1.01	0.25

^1^ Based on PhNTs, whose values are identical with the efficiency values. ^2^ TON = [Substrate]_0_/[1]_0_. ^3^ TOF = TON/h.

**Table 6:** The yield and selectivity of products (benzaldehyde, styrene oxide, and 2-phenyl-1-tosylaziridine for the catalytic oxidation of styrene in MeCN at 323 K in the presence of water, D_2_O, or a buffer. [1]_0_ = 1 × 10^−3^ M, [PhINTs]_0_ = 1 × 10^−1^ M, [styrene]_0_ = 3 × 10^−1^ M, [H_2_O, D_2_O]_0_ = 1.5 × 10^−2^ M, and [buffer]_0_ = 2 × 10^−1^ mL under air.

Entry	MeCN-X	[X]_0_ (10^−3^ M)	Yield (%) ^1^			Selectivity (%)			TON ^2^	TOF (1/h) ^3^
	X=		BZ	SO	SNTs	BZ	SO	SNTs		
1	-	-	18.2	14.65	4.16	49	40	11	37.01	9.25
2	H_2_O (pH 7)	15	8.62	6.13	1.23	54	38	8	15.98	3.99
3	H_2_O	50	7.89	5.34	0.65	57	38	5	13.88	3.47
4	H_2_O	100	5.11	3.49	0.32	57	39	4	8.92	2.23
5	H_2_O	200	3.79	2.61	0.15	58	40	2	6.55	1.64
6	H_2_O (pH 4.7)	15	16.52	26.17	6.72	33	53	14	49.41	12.35
7	H_2_O (pH 8)	15	5.95	-	-	100	-	-	5.95	1.49
8	D_2_O	15	5.12	3.86	0.75	53	40	7	9.73	2.43
9	H_2_O^18^	15	9.21	7.18	0.92	53	41	5	17.31	4.32

^1^ Based on PhNTs, whose values are identical to the efficiency values. ^2^ TON = [Substrate]_0_/[1]_0_. ^3^ TOF = TON/h.

**Table 7:** The yield and selectivity of products (Bz, benzaldehyde; SO, styrene oxide; and SNTs, 2-phenyl-1-tosylaziridine) for the catalytic oxidation of styrene with different *para*-substituted pyridines in MeCN at 323 K. [1]_0_ = 1 × 10^−3^ M, [PhINTs]_0_ = 1 × 10^−1^ M, [styrene]_0_ = 3 × 10^−1^ M, and [*para*-substituted pyridine]_0_ = 1 × 10^−2^ M under air.

Entry	Co-Ligand	Yield (%)			Selectivity (%)			TON	TOF (1/h)
	4R-S	BZ	SO	SNTs	BZ	SO	SNTs		
1	4-Me-pyridine	24.16	17.915	5.11	51	38	11	47.18	11.79
2	pyridine	31.15	20.02	6.32	54	35	11	57.49	14.37
3	4-C(O)C_6_H_5_-pyridine	34.28	21.76	8.53	53	34	13	64.57	16.14
4	4-C(O)CH_3_-pyridine	35.467	22.43	9.31	53	34	13	67.21	16.80
5	4-CN-pyridine	39.43	25.75	11.36	51	34	15	76.54	19.13

**Table 8:** Redox potential data of **1**, **1**/PhIO, and **1**/PhINTs adducts.

Entry	Complex	*E*_pa_ (Fe^3+/2+^) (V) vs. SCE	*E*_pc_ (Fe^3+/2+^) (V) vs. SCE	*E*_1/2_ (Fe^3+/2+^) (V) vs. SCE
1	[Fe^II^(PBI)_3_](OTf)_2_	+0.934	+0.870	+0.902
2	[Fe^II^(PBI)_3_](OTf)_2_ + PhIO	−0.076	−0.153	−0.115
3	[Fe^II^(PBI)_3_](OTf)_2_ + PhINTs	−0.130	−0.213	−0.171

**Table 9:** Kinetic data for the stoichiometric oxidation of styrene with (PBI)Fe^III^(PhINTs) in different solvents.

Entry	Solvent	[S]_0_ (M)	T (K)	*k*′_obs_ (10^−3^ s^−1^)	σ	TE	*k*_2_ (10^−3^ M^−1^ s^−1^)
1	MeCN	0.3	293	1.29			4.3
2	MeCN	0.5	293	3.69	0	1.34	7.38
3	MeCN	0.6	293	4.67			7.78
4	MeCN	0.9	293	5.67			6.3
5	MeCN	1.5	293	11.18			7.45
6	MeCN	1.5	288	6.4			4.26
7	MeCN	1.5	298	36.3			24.2
8	MeCN	1.5	303	62.7			41.8
9	CF_3_CH_2_OH	0.3	293	0.57			1.9
10	CF_3_CH_2_OH	0.5	293	1.26	0	1.34	2.52
11	CF_3_CH_2_OH	0.6	293	1.81			3.02
12	CF_3_CH_2_OH	0.9	293	2.42			2.68
13	CF_3_CH_2_OH	1.5	293	3.77			2.51
14	CF_3_CH_2_OH	1.5	288	1.63			1.09
15	CF_3_CH_2_OH	1.5	298	4.57			3.05
16	CF_3_CH_2_OH	1.5	303	6.33			4.22

*k_sd_* = 0.0002 s^−1^ (293 K); 0.00017 s^−1^ (288 K); 0.00024 (298 K); 0.00027 (303 K).

**Table 10:** Kinetic data for stoichiometric oxidation of *para*-substituted styrenes with **1**/PhINTs adduct in different solvents at 293 K.

Entry	Substrate	Solvent	[S]_0_ (M)	*k*′_obs_ (10^−3^ s^−1^)	σ	TE	*k*_2_ (10^−3^ M^−1^ s^−1^)
1	styrenes	MeCN	0.5	3.69	0	1.34	7.38
2	4-methoxystyrene	MeCN	0.5	183	−0.27	1.1	366
3	4-methylstyrene	MeCN	0.5	29.3	−0.17	0.41	58.6
4	4-chlorostyrene	MeCN	0.5	11.9	0.23	0.23	23.8
5	4-cyanostyrene	MeCN	0.5	88.8	0.66	0.93	177.6
6	styrene	CF_3_CH_2_OH	0.5	1.26	0	1.34	2.52
7	4-methoxystyrene	CF_3_CH_2_OH	0.5	57.53	−0.27	1.1	115.06
8	4-methylstyrene	CF_3_CH_2_OH	0.5	5.47	−0.17	0.41	10.9
9	4-chlorostyrene	CF_3_CH_2_OH	0.5	2.2	0.23	0.23	4.4
10	4-cianostyrene	CF_3_CH_2_OH	0.5	21.9	0.66	0.93	43.8

## Data Availability

Data are contained within the article.
